# Marine picocyanobacterial PhnD1 shows specificity for various phosphorus sources but likely represents a constitutive inorganic phosphate transporter

**DOI:** 10.1038/s41396-023-01417-w

**Published:** 2023-04-22

**Authors:** Bhumika S. Shah, Benjamin A. Ford, Deepa Varkey, Halina Mikolajek, Christian Orr, Vitaliy Mykhaylyk, Raymond J. Owens, Ian T. Paulsen

**Affiliations:** 1 School of Natural Sciences, Macquarie University, Sydney, NSW Australia; 2 ARC Centre of Excellence in Synthetic Biology, Macquarie University, Sydney, NSW Australia; 3 Diamond Light Source Ltd., Harwell Science and Innovation Campus, Didcot, UK; 4 Division of Structural Biology, The Wellcome Centre for Human Genetics, University of Oxford, Oxford, UK; 5 Structural Biology, Rosalind Franklin Institute, Harwell Science and Innovation Campus, Didcot, UK

**Keywords:** Environmental microbiology, Comparative genomics, Structural biology

## Abstract

Despite being fundamental to multiple biological processes, phosphorus (P) availability in marine environments is often growth-limiting, with generally low surface concentrations. Picocyanobacteria strains encode a putative ABC-type phosphite/phosphate/phosphonate transporter, *phnDCE*, thought to provide access to an alternative phosphorus pool. This, however, is paradoxical given most picocyanobacterial strains lack known phosphite degradation or carbon-phosphate lyase pathway to utilise alternate phosphorus pools. To understand the function of the PhnDCE transport system and its ecological consequences, we characterised the PhnD1 binding proteins from four distinct marine *Synechococcus* isolates (CC9311, CC9605, MITS9220, and WH8102). We show the *Synechococcus* PhnD1 proteins selectively bind phosphorus compounds with a stronger affinity for phosphite than for phosphate or methyl phosphonate. However, based on our comprehensive ligand screening and growth experiments showing *Synechococcus* strains WH8102 and MITS9220 cannot utilise phosphite or methylphosphonate as a sole phosphorus source, we hypothesise that the picocyanobacterial PhnDCE transporter is a constitutively expressed, medium-affinity phosphate transporter, and the measured affinity of PhnD1 to phosphite or methyl phosphonate is fortuitous. Our MITS9220_PhnD1 structure explains the comparatively lower affinity of picocyanobacterial PhnD1 for phosphate, resulting from a more limited H-bond network. We propose two possible physiological roles for PhnD1. First, it could function in phospholipid recycling, working together with the predicted phospholipase, TesA, and alkaline phosphatase. Second, by having multiple transporters for P (PhnDCE and Pst), picocyanobacteria could balance the need for rapid transport during transient episodes of higher P availability in the environment, with the need for efficient P utilisation in typical phosphate-deplete conditions.

## Introduction

Phosphorus (P) is an essential biological building block, integral for processes such as energy use (ATP), cell structure (phospholipids), and storage of genetic information in nucleic acids [1]. Dissolved P is also vital to the biogeochemistry of marine environments. The availability of the primary bioavailable form of P in its most oxidised state (+5), the inorganic phosphate ion (P_i_), significantly influences the growth, abundance, and diversity of the most abundant photosynthetic microorganisms on Earth, marine picocyanobacteria of the genera *Prochlorococcus* and *Synechococcus* [2–4]. Low-nanomolar concentrations of P_*i*_ have been reported in various marine environments, which could limit picocyanobacterial growth in these areas [5].

Marine picocyanobacteria have a well-characterised response to P_*i*_ limitation [5–7], leading to significant alterations in gene expression, particularly of the phosphate regulation (*Pho* regulon) network. The nature and organisation of the *Pho* regulon is highly variable, even between related picocyanobacteria [7], corresponding to different adaptation strategies between different ecotypes [4, 6]. Genes encoding the high-affinity multicomponent periplasmic binding protein-dependent P_*i*_ specific transporter (Pst) are shown to be significantly up-regulated under P_*i*_ starvation in marine picocyanobacteria [7]. Marine picocyanobacterial genomes lack genes encoding the low-affinity constitutively expressed single-component P_*i*_ symporter system (Pit) found in *E. coli* [4, 8, 9]. It is hypothesised that P_*i*_ uptake via the Pit system could be energetically more favourable due to the symport with protons at 1:1 stoichiometry as opposed to P_*i*_ uptake via the Pst system that potentially requires hydrolysis of two ATP per substrate transported [10].

Picocyanobacterial strains also encode a well-conserved predicted ABC-type phosphite/phosphate/phosphonate transporter, PhnD_1_CE, which is thought to provide access to an alternative P pool such as organic phosphonates, Pn (P valence +3) or the inorganic reduced P compound phosphite, P_t_ (P valence +3) [5]. The ability to uptake alternate P sources could provide significant competitive advantages under P_*i*_-depleted conditions. Physiological studies on picocyanobacteria, however, demonstrate variability in the expression of the PhnD_1_CE transport system under P stress. While some picocyanobacterial strains induce the expression of PhnD_1_CE transporter in a P-deficient media as shown for *Synechococcus* WH8102 [11], in some *Prochlorococcus* strains, it is not induced under P limitation [12] or can be constitutively expressed [11]. Several picocyanobacterial strains also encode an additional PhnCD_2_E transporter that clusters distinctly on protein phylogenetic trees [2, 3].

As the periplasmic substrate-binding component of the two predicted picocyanobacterial Pn transporters, PhnD1 and PhnD2, are typically co-expressed with the PhnC and PhnE components, the binding proteins have been used as proxies to investigate the substrate specificities of the respective transporter to understand their role in P acquisition [2, 3, 13]. Biophysical and structural data on PhnD1 and PhnD2, isolated from *Prochlorococcus* MIT9301, show diverse substrate specificity. While *Prochlorococcus* MIT9301_PhnD1 shows a high affinity for P_t_ with a binding affinity (K_*D*_) in the sub-micromolar range and comparatively weaker affinity to P_i_ and methylphosphonate (MPn), *Prochlorococcus* PhnD2 show a stronger affinity for MPn followed by P_t_ [2, 3, 13]. Unlike the *Prochlorococcus* PhnD proteins, there is a distinct lack of understanding regarding the specificity range of PhnD proteins within *Synechococcus* strains.

For various *Synechococcus* isolates, low P quotas and high uptake rates underscore the intense P competition in nutrient deplete oligotrophic environments [5, 14]. Low available P concentrations exert an intense selective pressure that influences the repertoire of P acquisition mechanisms of the related *Prochlorococcus* strains [12], most of which are also shared by *Synechococcus* isolates [5]. Several genes responsible for P acquisition have been acquired horizontally [12], indicating acquisition strategies between different strains likely reflect local P availability.

*Synechococcus* strains from clade I (e.g. CC9311) and IV generally co-occur in coastal and/or temperate mesotrophic open ocean waters, largely above 30^°^N and below 30^°^S [15, 16], alongside a broad nitrate and phosphate concentration range (0.03 to 14.5 µM and 0.2 to 1.2 µM, respectively) [5]. In contrast, *Synechococcus* clade II strains (e.g. CC9605) are abundant in coastal/continental shelves strictly in the subtropical/tropical latitudes (between 30^°^N and 30^°^S) [15–18]. *Synechococcus* clade III isolates (e.g. WH8102) do not show any latitudinal preference but are restricted to limited nitrate and phosphate concentrations [15], whereas clades CRD1 (e.g. MITS9220) and CRD2 are most successful in low Fe waters [19]. We examined PhnD1 from four marine *Synechococcus* strains (CC9311, CC9605, WH8102 and MITS9220) isolated from diverse environmental niches to characterise their corresponding ligand binding preferences and to explore the relationship between PhnD1 ligand specificity range and ecological niches.

## Results and discussion

### PhnD1 proteins in picocyanobacteria are lineage partitioned

**Fig. 1:**
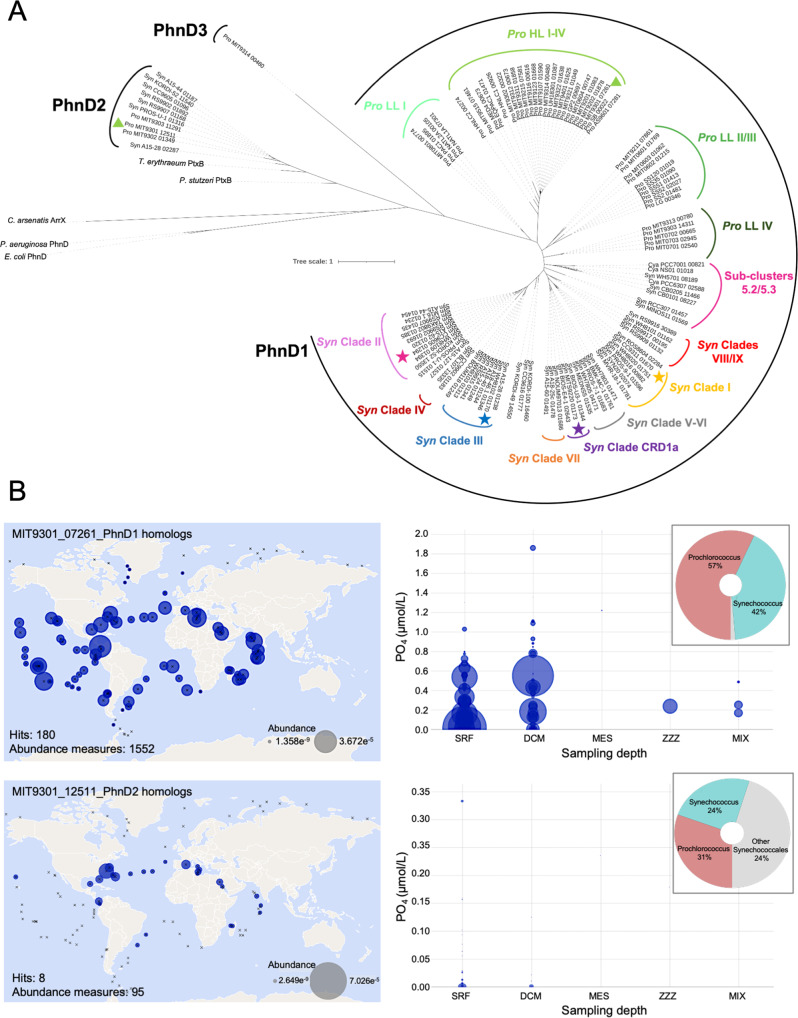
Phylogenetic and genomic analyses of picocyanobacterial PhnD1 sequences. **A** The amino acid sequences of *Synechococcus (Syn)* and *Prochlorococcus (Pro)* PhnD1 (CK_860), PhnD2 (CK_6203) and PhnD3 (CK_56876), as well as previously characterised non-picocyanobacterial PhnD proteins were aligned using MAFFT [40], and the phylogenetic tree inferred using IQ-Tree [41]. The final phylogenetic tree was visualised using iTOL [42]. The four *Synechococcus* PhnD1 proteins (CC9311, CC9605, WH8102 and CRD1a) characterised in this study are highlighted on the tree. Genes are numbered according to their Cyanorak cluster designation [20]. **B** The environmental abundance for *Prochlorococcus* MIT9301_PhnD1 (top-left) and MIT9301_PhnD2 (bottom-left) picocyanobacterial homologues extracted from the Tara Oceans MetaT dataset [57]. The transcript abundance is plotted for surface waters, with a circle size corresponding to the measured abundance at a particular sampling site. Sampling sites are denoted by an ‘X’. The corresponding bubble plot (right) for the identified transcripts across sampling depths (SRF, surface waters; DCM, deep chlorophyll maximum; MES, mesopelagic zone; MIX, marine epipelagic mixed layers;) is depicted as a function of measured phosphate concentration. The Krona plot in the inset shows the taxonomic distribution of MIT9301_PhnD1, and MIT9301_PhnD2 homologues selected to analyse the metatranscriptome abundance.

The predicted phosphonate-binding protein (PhnD1) under study is found within the genome of all 97 sequenced picocyanobacteria strains in the Cyanorak database, comprising cluster CK_860 [20]. While the gene encoding PhnD1 is highly conserved among all picocyanobacterial genomes, our phylogenetic tree reveals it is distinctly partitioned by lineage, with *Prochlorococcus* representatives group separately from *Synechococcus* isolates and each clustering into clade-level groups (Fig. 1A). This highlights that the PhnD1 gene has been retained across evolutionary pressures and likely propagated by vertical transfer rather than horizontal gene transfer events. The latter is unlike the predicted duplicate copies, PhnD2 (cluster CK_6203; also annotated as PtxB [2, 3]) present in only a small number of strains (*Prochlorococcus* HLII/LLIV and *Synechococcus* clade II isolates) and a single PhnD3 (cluster CK_56876; only found in *Prochlorococcus* MIT9314) that cluster remotely from the PhnD1 genes. The low conservation of PhnD2 and PhnD3 indicates these are likely laterally transferred or results of lineage-specific gene duplications among ecotypes in similar environmental niches.

To evaluate the distribution and expression of PhnD1 and PhnD2 in marine picocyanobacterial populations, we investigated the metagenomes and metatranscriptomes available from the Ocean Microbial Reference Gene Catalogue (OM-RGC) [21, 22]. For comparison, we provide distribution maps for *Synechococcus* and *Prochlorococcus* lineage-specific genes (Supplementary Fig. S1A, B). This analysis revealed that picocyanobacterial PhnD1 transcripts (Fig. 1B) and genes (Supplementary Fig. S1C, D) are not only enriched in mostly P_i_-replete waters but also some P_i_-deplete areas across several Pacific Ocean, Atlantic Ocean and Indian Ocean sites (surface water as well as the deep chlorophyll maximum zone). In contrast, picocyanobacterial PhnD2 homologues were most abundant in P_i_-limited surface waters in the North-western Atlantic Ocean, the Mediterranean Sea and the Gulf of Mexico.

Previous studies show that *Prochlorococcus* MIT9301, which encodes both *phnD1* and *phnD2*, can use P_t_ or MPn as the sole P source to support its growth [2, 3, 23]. The ability of *Prochlorococcus* MIT9301 to grow on P_t_ or MPn is attributed to the predicted phosphonate oxidative pathway encoded by the putative *phnY* (cluster CK_55307) and *phnZ* (cluster CK_7402) genes [23, 24] and a NAD-dependent phosphite dehydrogenase *ptxD* (cluster CK_56808), respectively. These predicted Pn and P_t_ utilisation genes are co-located with the *phnC*_*2*_*D*_*2*_*E*_*2*_ (also annotated as *ptxABC*) transport system in some picocyanobacterial strains. Based on these observations, we hypothesise that, by extension, all picocyanobacterial isolates encoding the *phnC*_*2*_*D*_*2*_*E*_*2*_ and the adjacent *phnY*, *phnZ* and *ptxD* genes have the potential to metabolise P_t_ or MPn, providing an ecological advantage to survive in a P_i_-depleted environment. Our comparative genome analysis reveals *Prochlorococcus* and *Synechococcus* genomes sequenced to date, however, lack the *phnY*, *phnZ* and *ptxD* genes, suggesting these strains may not be able to utilise P_t_ or MPn.

Based on the in vitro studies showing a very high affinity of *Prochlorococcus* MIT9301 PhnD1 to P_t_ [3, 13], it is predicted that the transporter encoded by *phnD*_*1*_*C*_*1*_*E*_*1*_ could be a phosphite transporter. However, studies show that *Prochlorococcus* strains MIT9313 and MED4 (lacking the *phnC*_*2*_*D*_*2*_*E*_*2*_ transport system and the adjacent metabolising genes) are unable to grow and utilise P_t_ (or MPn) and instead are only able to utilise P_i_ for their growth [2, 3, 23]. Other studies examining the P-starvation response in picocyanobacteria indicate none of the components of the *phnD*_*1*_*C*_*1*_*E*_*1*_ transport system showed any significant upregulation in P-deplete conditions, as opposed to the strong induction of genes previously implicated in P-scavenging, such as the high-affinity *pstS* gene [7, 12].

We examined the *phnD*_*1*_*C*_*1*_*E*_*1*_ genome context and find a highly conserved gene (*tesA*; cluster CK_171), encoding a predicted lysophospholipase L1-like esterase, co-located in almost all picocyanobacterial sequenced genomes. While the function of the periplasmic TesA in picocyanobacteria is unclear, it has been previously proposed to be part of a complex enzymatic system responsible for phospholipid membrane homeostasis in *P. aeruginosa* [25]. Picocyanobacteria (*Prochlorococcus* MED4 and *Synechococcus* WH8102, WH7803, WH5701*)* are shown to have the ability to substitute sulfolipids for phospholipids in their membrane to economise P utilisation under P-limitation [26]. The same study also showed that while the “substitute lipids” are dominant in the P-limited cultures, the phospholipid substitution occurred in all cases, including in the P-replete conditions, although to a much smaller extent. The genes required for sulfolipid biosynthesis, *sqdB* (cluster CK_123) and *sqdX (*cluster CK_333), are conserved in all sequenced picocyanobacterial genomes and not just those abundant in the P-deplete conditions. It remains unclear if the conservation of sulfolipid biosynthesis genes (*sqdB, sqdX*) and the co-localisation of the *phnD*_*1*_*C*_*1*_*E*_*1*_ transporter with conserved *tesA* are correlated and what physiological advantage in terms of P utilisation this would provide to the marine picocyanobacterial strains. It is possible that TesA could play a role in breaking down phospholipids found in picocyanobacteria, such as the prevalent phosphatidylglycerol (PG). While the specific breakdown products of PG in cyanobacteria are currently unknown, in yeast, PG can be degraded to diacylglycerol and glycerol-3-phosphate (G3P) [27]. In this case, alkaline phosphatase could then cleave the phosphoryl group from the glycerol-3-phosphate, producing glycerol and inorganic phosphate (P_i_), which could be taken up by the PhnDCE or Pst transport system, setting the stage for a constant phosphorus supply irrespective of the environmental conditions.

### *Synechococcus* PhnD1 proteins show a preferential binding affinity for phosphite, phosphate and methylphosphonate

**Fig. 2:**
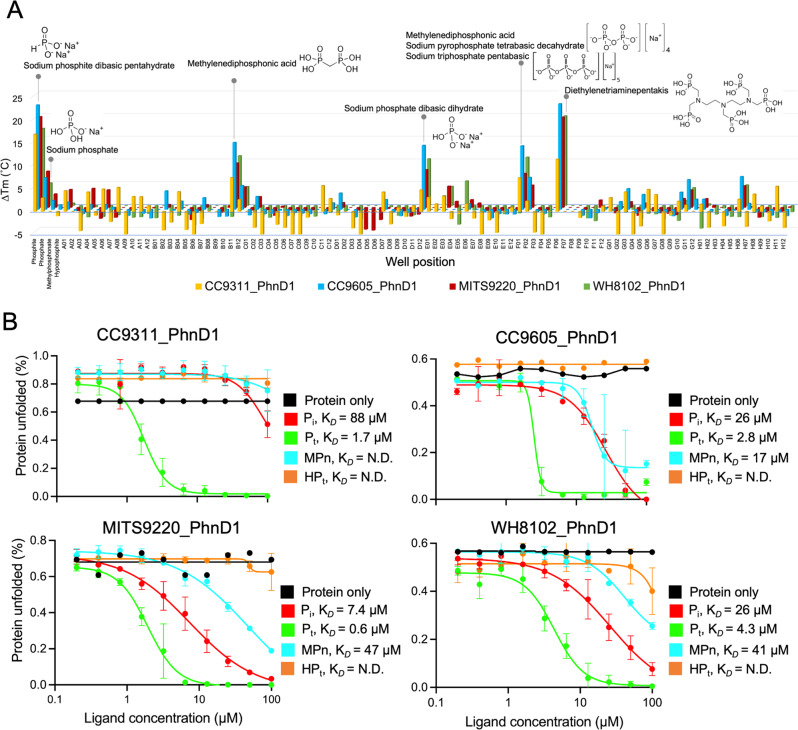
Ligand screening and binding affinity measurement of *Synechococcus* PhnD1 proteins. **A** DSF thermal melt assay was used to screen ligands for CC9311_PhnD1 (yellow), CC9605_PhnD1 (blue), MITS9220_PhnD1 (red) and WH8102_PhnD1 (green) proteins in the presence of a range of cocktail solutions (Silver Bullets 96-well screen) containing unique biologically relevant potential substrates as well as with various P sources (P_i_, P_t_, MPn and HP_t_). These data show that significant change in the melting temperature (ΔT_*M*_) is conferred only in cocktail conditions comprising P sources. **B** The measured binding affinity of the four *Synechococcus* PhnD1 proteins calculated based on the isothermal analysis of the DSF data (Supplementary Figs. S4–S7) upon incremental addition of the respective ligand; P_i_ (red), P_t_ (green), MPn (blue) or HP_t_ (orange) is depicted. Reported affinities were computed using the Python package [29] and plotted on GraphPad Prism.

Several studies have examined the P-binding specificities for different PhnD proteins in *Prochlorococcus* [2, 3, 13]. This is the first study to report the substrate specificity range of *Synechococcus* PhnD1 proteins. We expressed and purified four *Synechococcus* PhnD1 proteins (CC9311_PhnD1, CC9605_PhnD1, MITS9220_PhnD1 and WH8102_PhnD1; Supplementary Fig. S2) and evaluated their potential ligand binding preferences by differential scanning fluorimetry (DSF). This was done by measuring the increase in melting temperature (T_*M*_) of the protein in the presence of potential ligands. The first-pass ligand screen encompassed compounds (Supplementary Table S1), including a diverse range of biologically active small molecules, trace metals, and common metabolic nutrients (C, N, S, P sources) as well as inorganic phosphate (P_i_), phosphite (P_t_), methylphosphonate (MPn), and hypophosphite (HP_t_). This comprehensive DSF screen reveals (Fig. 2A) all four PhnD1 proteins have higher thermal stability (ΔT_*M*_ 5–23 °C) only in cocktail conditions comprising P sources. Among the single P-sources tested, the most significant shift in the T_*M*_ is observed in the presence of P_t_ (ΔT_*M*_ 17–23 °C), followed by P_i_ (ΔT_*M*_ 3–8 °C). A second DSF screen comprising 60 distinct individual P-sources were tested for CC9605_PhnD1 protein, including those representing the hydrophilic polar headgroups of various phospholipids such as 3-phospho glyceric acid, phosphoryl choline, inositol phosphate and phospho-serine (Supplementary Fig. S3). With the exception of carbamyl phosphate and phospho glycolic acid, which may act as a phosphate proxy due to their comparatively smaller size, no other phosphorous sources showed a marked increase in the thermal stability, indicating they are unlikely to be PhnD1 substrates.

A fluorometric isothermal approach [28, 29] was further utilised to determine the binding dissociation constants (K_D_) of the four *Synechococcus* PhnD1 proteins in the presence of P_i_, P_t_, MPn, and HP_t_ (Fig. 2B). This approach involves measuring incremental changes in the thermal stability (Supplementary Figs. S4–S7) caused by the partial formation of protein-ligand complexes, measured by an extrinsic dye [28, 29]. All four *Synechococcus* PhnD1 proteins displayed an affinity for at least two P sources: P_i_ and P_t_ (Fig. 2B); however, the specific binding affinities for these differ between each strain, as summarised in Table 1. Of the four proteins under study, MITS9220_PhnD1 displayed a comparatively stronger affinity for P_t_ and P_i_. The derived K_*D*_ value for P_t_ is at least an order of magnitude stronger than P_i_ for each PhnD1 protein, indicating a markedly stronger affinity for P_t_. This is intriguing, given the fact that most strains of picocyanobacteria lack known P_t_ utilisation proteins.

**Table 1:** Dissociation constants for *Synechococcus* PhnD1 proteins and their homologues.

Protein	Strain	Gene ID	Clade	Ecological conditions	P_i_^a^ (μM)	P_t_^a,b^ (μM)	MPn^a^ (μM)	2-AEP ^a,b^ (μM)	Affinity measurement	Ref.
CC9311_PhnD1	*Syn sp*. CC9311	01670	I	Coastal and/or temperate mesotrophic open ocean [19]	88 ± 20	1.7 ± 0.9	*–*	_*NM*_	nanoDSF	Our study
CC9605_PhnD1	*Syn sp*. CC9605	01294	II	Offshore, oligotrophic tropical or subtropical waters [19]	26 ± 10	2.8 ± 2	17 ± 3	_*NM*_	nanoDSF	
MITS9220_PhnD1	*Syn sp*. MITS9220	01173	CRD1a	Low iron, high nutrient equatorial upwelling regions [58, 59]	7.4 ± 1.3	0.67 ± 0.1	47 ± 9	_*NM*_	nanoDSF	
WH8102_PhnD1	*Syn sp*. WH8102	01170	III	Ultraoligotrophic open-ocean waters [19]	26 ± 8	4.3 ± 0.5	41 ± 5	_*NM*_	nanoDSF	
Pm_PhnD1	*Pro*. MIT9301	07261	HLII	Strongly stratified surface waters, mainly tropical and subtropical regions [60]	54.9	0.12	39.0	*–*	ITC	[3]
					182 ± 26	0.051 ± 0.004	109 ± 8	*–*	Microscale thermophoresis	[13]
Pm_PtxB^*c*^	*Pro*. MIT9301	12511	HLII	Strongly stratified surface waters, mainly tropical and subtropical regions [60]	*–*	2.0	0.8	*–*	ITC	[3]
Ec_PhnD	*E. coli* UTI89	C4699	n/a	n/a	50	_*NM*_	1.3	<0.05	Intrinsic fluorescence	[61]
					*–*	*–*	18.4	0.1	ITC	[3]

^a^_*NM*_ denotes binding not measured.

^b^Dash indicates binding not detected in the respective study.

^c^PtxB is annotated as PhnD2 in the cyanorak database.

Except for the CC9311_PhnD1, isolated from the strain found in the mesotrophic marine settings, three *Synechococcus* PhnD1 proteins also show a measurable affinity for MPn: CC9605_PhnD1 (17 ± 3 μM), WH8102_PhnD1 (41 ± 5 μM), and MITS9220_PhnD1 (47 ± 9 μM). While the binding affinity of these PhnD1 proteins for MPn is comparatively weaker than P_i_ and P_t_, the measured binding constants are comparable to that known for the *Prochlorococcus* MIT9301 PhnD1 homologue (Pm_PhnD1, K_*D*_ = 39–108 μM) (21). As organic phosphonates arise from cellular components, such as phospholipids, nucleic acids, amino acids, and polysaccharides, this broad substrate affinity observed for some *Synechococcus* isolates could potentially reflect an ability to access more labile forms of phosphorus [3, 30] rather than solely inorganic sources, which are generally sparingly soluble. As discussed earlier, none of the four Synechococcus strains under study (CC9311, CC9605, WH8102, MITS9220) harbours currently known putative P_t_ or MPn utilisation genes (*ptxD, phnY*/*phnZ* or C-P lyase) in their genome. Therefore, the affinity of *Synechococcus* PhnD1 proteins to P_t_ or MPn, analogous to the *Prochlorococcus* PhnD1 [3, 13], may be incidental, requiring further investigations into the structural mechanism of P selectivity for picocyanobacterial PhnD1 proteins.

### The crystal structure of MITS9220_PhnD1 in complex with phosphate reveals an extensive hydrogen bond network

**Fig. 3:**
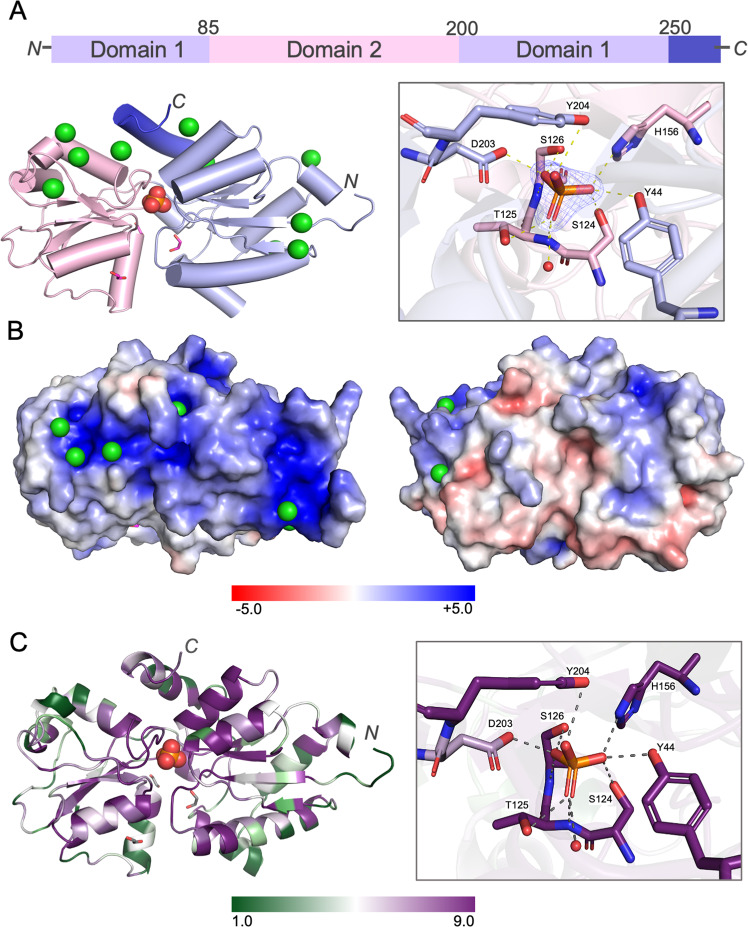
Crystal structure of MITS9220_PhnD1 in complex with phosphate. **A** Block diagram representation (top) of the primary structure of MITS9220_PhnD1 showing the two lobes and the amino acids that determine the border of each domain. A cartoon representation (bottom-left) of the overall fold of MITS9220_PhnD1 highlighting the two α/β domains; lobe 1 (violet) and lobe 2 (pink), with P_i_ drawn as spheres (red). Also included in the representation are ten Cl^−^ ions (green spheres) and 3 EDO (magenta sticks) molecules sequestered from the crystallisation condition. A detailed view of the ligand-binding pocket (bottom-right), outlining the interactions between P_i_ and MITS9220_PhnD1. The interacting sidechains and P_i_ are shown as sticks and coloured as per the respective domains, with the protein backbone shown as a cartoon. The single buried water is shown as a red sphere. **B** The electrostatic surface potential of the front (left) and back (right) of the MITS_9220 PhnD1 structure, with positive (blue) and negative charge (red) shown. **C** The protein sequence conservation mapping of picocyanobacterial PhnD1 homologues on MITS9220_PhnD1 structure (left) and ligand-binding pocket (right). The areas with high conservation (purple) and low conservation (green) are colour-coded.

While crystal structures of PhnD1 in complex with P_t_ and MPn from *Prochlorococcus* are available, there are no crystal structures of picocyanobacterial PhnD1 protein in complex with P_i_. Therefore, to understand the molecular mechanism of P_i_ selectivity, we determined a high-resolution crystal structure (2.0 Å) of *Synechococcus* MITS9220_PhnD1 with bound P_i_, resulting in a closed ligand-bound complex (Fig. 3). Data collection and final refinement statistics for the crystal structure are outlined in Table 2. The overall structure of MITS9220_PhnD1 is similar to that of other class II substrate binding proteins (SBPs) [31] and specifically to cluster-F SBPs [32, 33], with two α/β domains separated by a longer central hinge region (8–10 amino acids). The extended hinge region in cluster-F SBPs is believed to provide greater flexibility between the ligand-bound (closed) and unbound (open) conformations of cluster-F SBPs [33]. This hinge region encompasses a buried ligand-binding cavity and includes a P_i_ molecule within the MITS9220_PhnD1 structure copurified with the protein. The MITS9220_PhnD1 structure displays an unusual asymmetric distribution of electrostatic surface potential (Fig. 3B), where the front is completely enveloped by positive charge, attracting several Cl^-^ anions from the crystallisation buffer. While the presence of Cl^-^ on the surface might be a crystallisation artifact, the strong positively charged front may have functional significance in facilitating picocyanobacterial PhnD1 association with the predominantly negatively charged phospholipid membrane surface.

**Table 2:** Data collection and refinement statistics of MITS9220_PhnD1 structure.

Data collection				
Wavelength (Å)	3.0996	2.7552	5.166	4.8621
Beamline	I23	I23	I23	I23
Resolution (À)	106.61–2.02 (2.07–2.02)	101.2–1.8 (1.84–1.80)	106.4–3.37 (3.64–3.37)	106.4–3.39 (3.39–3.17)
Space group	C 1 2 1	C 1 2 1	C 1 2 1	C 1 2 1
Unit cell (a, b, c, α, β, γ)	63.6, 40.7, 106.7, 90, 92.1, 90	64.0, 40.7, 101.2, 90, 91.6, 90	63.9, 40.7, 106.4, 90, 92.2, 90	64.0. 40.8, 106.5 90, 92.2, 90
Total reflections^a^	384295 (9895)	884316 (14521)	14348 (1656)	17287 (1701)
Unique reflections^a^	18101 (1281)	24534 (1308)	3436 (552)	4119 (578)
Multiplicity^a^	21.2 (7.7)	36 (11.1)	4.2 (3)	4.2 (2.9)
Completeness (%)^a^	99.7 (95.6)	98.3 (89.3)	85.1 (67.8)	85.3 (68.2)
Mean I/*σ* (I)^a^	40.5 (12.2)	10.8 (1.1)	8.8 (5.6)	13.2 (8.2)
CC_half_^a^	1.000 (0.994)	0.999 (0.882)	0.976 (0.939)	0.989 (0.978)
R_merge_^a^	0.061 (0.113)	0.185 (1.19)	0.137 (0.202)	0.088 (0.116)
R_meas_^a^	0.062 (0.121)	0.187 (1.246)	0.158 (0.246)	0.101 (0.141)
R_pim_^a^	0.012 (0.042)	0.027 (0.346)	0.075 (0.137)	0.047 (0.077)
***Refinement***	**(PDB: 7S6G)**			
R_factor_/R_free_	0.155/0.1960			
RMSD bonds	0.0145			
RMSD angles	1.85			
*No. of non-H atoms*				
Protein	2129			
Ligands	17			
Ions	10			
Water	256			
Protein residues	273 (1 chain)			
*Average B-factors*				
Main/side chain	17.1/21.2			
Ligands/ions	30.4/48.5			
solvent	30.6			
*Ramachandran*				
favoured (%)	98.17			
allowed (%)	1.1			
Molprobity/clash score	0.72/0.69			

^a^Values in the parenthesis are for data in the high-resolution outer shell.

Even though clear electron density is observed in the substrate binding site, which appears to have a tetrahedral geometry consistent with the P_i_ molecule in the binding pocket (Fig. 3), all ligands under study (P_i_, P_t_, and MPn) have similar chemical structures. Therefore, careful assignment of P_i_ to the positive difference Fourier electron density was ensured by considering van der Waals contacts and hydrogen bonds in the most appropriate chemical orientation and comparing refinements with the P_i_ replaced by P_t_ and MPn (Supplementary Fig. S8). Additionally, we also collected X-ray data above (2550 eV) and below (2400 eV) the sulphur edge, which assisted in dismissing the remote possibility of a sulphate ion instead being sequestered within the binding cavity (Supplementary Fig. S9).

Within the crystal structure of MITS9220_PhnD1, the four P_i_ oxygens are bound by a total of 10 direct hydrogen bonds, forming an extensive H-bond network to the main chain and sidechains of residues distributed between the two domains (Fig. 3A). These include a tyrosine (Y44) at the beginning of α2; an -STS- motif (S124, T125, S126) in a loop region joining β6 and α4; a histidine (H156) at the beginning of α6; an H-acceptor aspartic acid (D203) and a capping tyrosine residue (Y204) found just before β10. A single water molecule buried deep within the cavity also contributes to this hydrogen bond network. This water molecule is further stabilised by intramolecular hydrogen bonds to residues on domain 1 (T65) and domain 2 (S123), contributing to the interactions that connect the two domains and occluding the binding pocket from the solvent. Similarly, the capping Y204 sidechain is engaged in hydrogen bonds to residues D11 (domain 1) and N174 (domain 2), contributing to the two-domain interactions.

Sequence analysis shows strong conservation of all key MITS9220_PhnD1 P_i_ binding residues among most picocyanobacteria PhnD1 homologues (Fig. 3C), suggesting an analogous P_i_ binding mechanism in these isolates. The only exception is a substitution of aspartic acid (D203) to a functional homologue asparagine residue in the three Cyanobium strains, all *Prochlorococcus* LL1 strains (MIT0901, NatL1A, NatL2A, PAC1), and most *Synechococcus* clade I strains (Syn MV1R-18, Syn20, Pros 9-1, WH8016, WH8020) available on the Cyanorak database. In the absence of the biophysical data, it is unclear if the Asp to Asn substitution at the binding site impacts the P specificity of PhnD1 in these isolates, given the essential role of D203 as an H-bond acceptor.

### MITS9220_PhnD1 structural comparison reveals the molecular basis of broad P specificity and medium P_i_ affinity

**Fig. 4:**
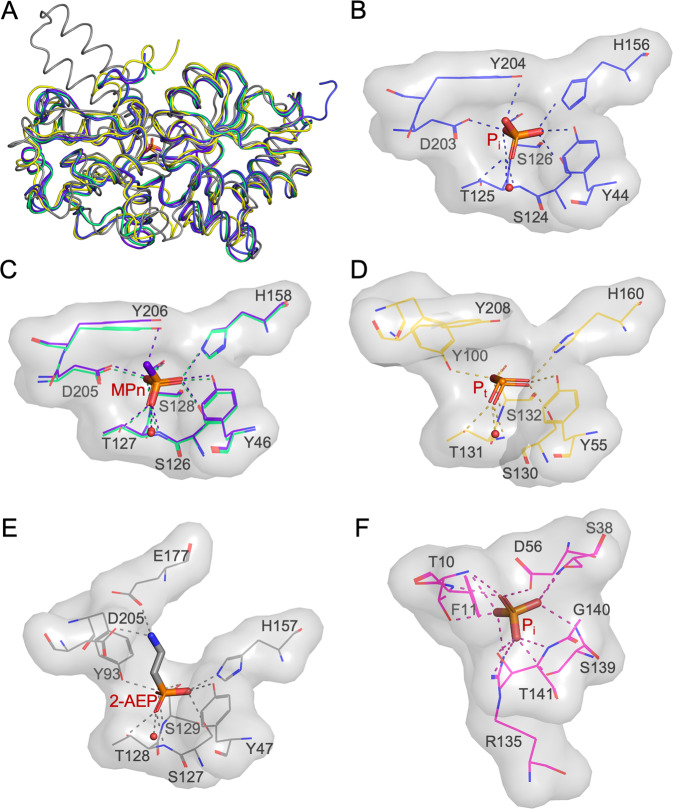
Comparison of MITS9220_PhnD1 protein structure with structural homologues. **A** The overlay of the crystal structure of *Synechococcus* MITS9220_PhnD1 in complex with P_i_ (blue; PDB 7S6G), *Prochlorococcus* MIT9301_PhnD1 (green; PDB 5LQ5) and MPn (purple; PDB 5LQ8), *Prochlorococcus* MIT9301_PhnD2 (yellow; PDB 5LV1). The ligand binding cavity (grey surface) with side-chain and ligand interactions is depicted for (**B**) *Synechococcus* MITS9220_PhnD1 in complex with P_i_, (**C**) *Prochlorococcus* MIT9301_PhnD1 and MPn, (**D**) *Prochlorococcus* MIT9301_PhnD2, (**E**) *E. coli* PhnD in complex with 2-AEP (grey; PDB 3P7I) and (**F**) *E. coli* PstS in complex with P_i_ (pink; PDB 2ABH).

A search for structural homologues using the Dali server [34] reveals many structures similar to MITS9220_PhnD1 bound to P_i_ (Table 3). Among them, the structure of Pm_PhnD1 (56% sequence identity) in complex with P_t_ and MPn is most similar to MITS9220_PhnD1, with an r.m.s.d. of 1.1 Å. The structure of Pm*_*PhnD2 in complex with P_t_, sequence identity of 30% to MITS9220_PhnD1, is the second closest structural homologue (r.m.s.d 1.7 Å). In comparison, the *E. coli* PhnD structure differs markedly with an r.m.s.d. of 2.4 Å, whereas the high-affinity P_i_-binding PstS protein in *E. coli* aligns distantly with an r.m.s.d. of 5.1 Å. The overall fold of *Synechococcus* MITS9220_PhnD1 (with P_i_) structure and the close structural homologues from *Prochlorococcus* MIT9301 [13] are very similar (Fig. 4A). The chemical structure of P_i,_ P_t_, and MPn only differs at the R1 position substituted by OH, H and CH_3_, respectively. In all these structures, the R1 position of the respective P-ligand is capped by a tyrosine residue (Fig. 4B–D), leading to a smaller binding pocket for picocyanobacterial PhnD1 and PhnD2 proteins. In contrast, the *E. coli* PhnD (Ec_PhnD) crystal structure shows two H-bond acceptors, D205 and E177, in the active site, resulting in a comparatively larger binding cavity that can accommodate bulkier phosphonates, such as 2-aminoethylphosphonate (2-AEP) (Fig. 4E). The structural comparisons thus reveal the significance of the capping tyrosine residue as a steric barrier and rationalise why picocyanobacterial strains in the past were unable to bind and metabolise large complex Pn, such as 2-AEP [2, 23] but instead only use simple phosphonates such as MPn as a sole P source in strains encoding the potential Pn utilisation genes (*phnY*/*phnZ*) [23].

**Table 3:** Sequence and structural similarities of MITS9220_PhnD1 with homologous SBPs.

Protein	Seq. ID (%)	PDB	r.m.s.d. (Å)	Ligand	Resolution (Å)	Reference
Pm_PhnD1	56	5LQ5	1.1	P_t_	1.46	[13]
		5LQ8	1.1	MPn	1.52	
Pm_PtxB (PhnD2)	30	5LV1	1.7	P_t_	2.12	[13]
*T. erythraeum IMS101* PtxB	27	5JVB	1.6	P_t_	1.95	[13]
		5LQ1	1.7	MPn	1.41	
*P. stutzeri* PtxB	32	5O2J	1.9	P_t_	1.52	[13]
		5O37	2.0	MPn	1.37	
		5O2K	5.1	no ligand	2.10	
Ec_PhnD	22	3P7I	2.4	2-AEP	1.7	[62]
		3QUJ	2.4	unknown ligand	2.2	
*P. aeruginosa* PhnD	21	3N5L	2.3	Unknown	1.97	n/a
*C. arsenatis* ArrX	22	6X6B	2.4	Sulphate	1.67	[63]
		6XL2	2.5	Arsenate	1.74	
		6XAD	2.6	Formate	1.89	
		6XAB	2.7	Acetate	1.78	
		6X9G	3.1	Malonate	1.68

As discussed earlier, the P_i_ in MITS9220_PhnD1 structure is co-ordinated by ten hydrogen bonds (Fig. 4B), where the three oxygen atoms are engaged in multiple H-bonds, three each with O1, O3 and O4 in a trigonal geometry. The O2 atom (representing the R1 group) is, however, stabilised by a single H-bond formed by the capping residue Y204 (Supplementary Table S3). In contrast, the crystal structures of the high-affinity P_i_-specific binding protein PstS, for example, in *E. coli* (PDB 2ABH), show an extensive hydrogen bond network [35, 36] consisting of 14 H-bonds (Fig. 4F). In addition to the three oxygen atoms O1, O3 and O4 engaging in three H-bonds each, the O2 atom in the case of *E. coli* PstS is stabilised by five H-bonds in a pentagonal geometry, explaining the high-affinity and specificity of PstS to P_i_. The comparison of the binding pocket of the medium and high-affinity P_i_ binding proteins, MITS9220_PhnD1 and *E. coli* PstS, respectively, highlights the central role of the H-bond network in determining the specificity and binding affinity of the P-ligands. Our structural comparisons thus provide molecular insights into the potential fortuitous binding of P_t_ or MPn and the comparatively lower affinity of picocyanobacterial PhnD1 to P_i_.

Low-affinity substrate-binding proteins are better suited for rapid substrate turnover, while high-affinity substrate-binding proteins are well suited for substrate scavenging at low concentrations. By having multiple nutrient transporters with different binding affinities and transport rates, cells can mitigate a “rate-affinity trade-off” and ensure the efficient transport of nutrients under different conditions [37]. In picocyanobacteria, the high-affinity Pst transporter could therefore be used to maintain a steady supply of P_i_ under phosphate-deplete conditions, while the comparatively low-affinity Phn transporter with potentially fast transport rates could be used to import large amounts of P_i_ when it is transiently available. For example, in times of upwelling, riverine inputs or P_i_ bursts from plankton or viral lysis. This would thus allow the picocyanobacterial cells to balance the need for rapid transport of nutrients under occasional conditions of plenty, with the need to efficiently utilise available P resources under typical phosphate-deplete conditions.

### *Synechococcus* strains MITS9220 and WH8102 do not utilise P_t_ and MPn for growth as the sole P source

To test if *Synechococcus* strains not encoding *phnD2* and the adjacent P_t_/Pn utilisation genes can use alternative P sources other than P_i_, we examined their growth in culture conditions containing either P_t_ or MPn as the sole P source. We specifically selected *Synechococcus* strain MITS9220 (clade CRD1a representative) associated with mesotrophic environments and strain WH8102 (clade III representative) found in oligotrophic waters, to understand growth physiology for isolates from environments with known differences in P_i_ availability.

**Fig. 5:**
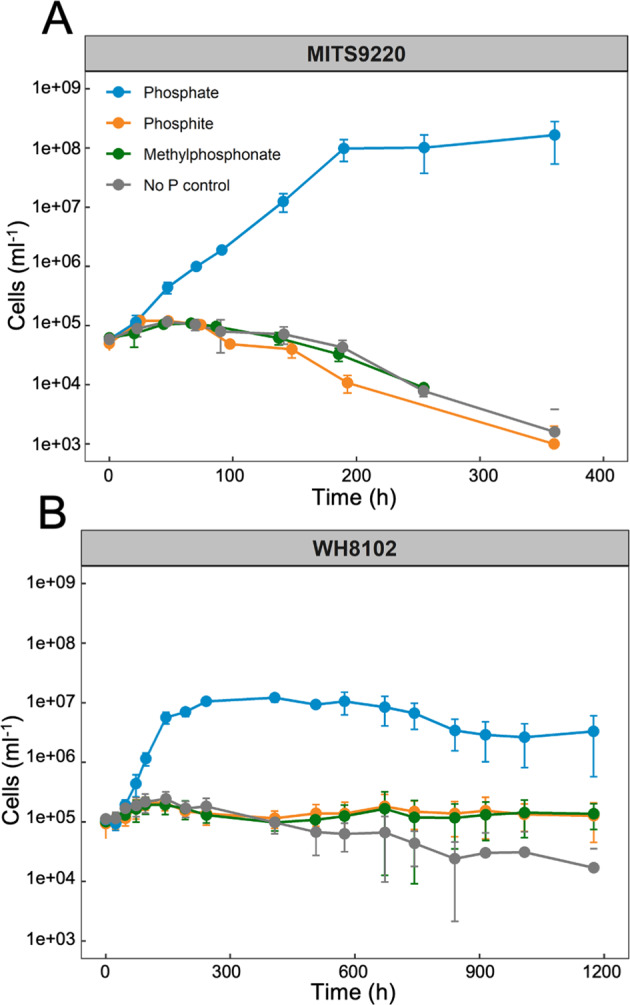
Growth curves of *Synechococcus* MITS9220 and WH8102 in the presence of various P sources as the sole P source. *Synechococcus* MITS9220 (**A**) and WH8102 (**B**) do not support well-defined exponential growth in the presence of P_t_ or MPn as a sole P source. The cultures were grown in PCR-S11 medium containing P_i_ (blue), P_t_ (orange), and MPn (green) in addition to the no P control (grey). The cell density was measured at regular intervals using a CytoFLEX S flow cytometer. The standard deviation of the mean cell density from triplicate cultures is represented as error bars.

We show both *Synechococcus* strains, MITS9220 and WH8102, when grown in the presence of either P_t_ or MPn as the sole P source, do not exhibit a clearly defined exponential growth (Fig. 5), as opposed to the growth seen in the presence of P_i_. It is possible that the *Synechococcus* strains tested may have lost their ability to utilise P_t_ or MPn due to prolonged exposure to P_i_-rich media in laboratory cultures. However, this scenario seems unlikely as the genomes of these strains lack currently known P_t_ or MPn metabolising genes (*ptxD, phnY*/*phnZ* or C-P lyase). A picocyanobacterial strain with these genes (e.g. *Prochlorococcus* MIT9301) is perfectly able to metabolise P_t_ and MPn as the sole P source to support its growth [2, 23], despite being in the laboratory cultures for an equivalent amount of time.

For *Synechococcus* MITS9220, beyond day 3, all non-phosphate culture conditions exhibited a steady decline in cell density, reaching an undetectable cell density after day 15, upon which the experiment was terminated for this strain (Fig. 5A). In contrast, while WH8102 did not show exponential growth, it was able to persist for much longer, with detectable cell densities measured for all culture conditions for the entire duration of the experiment (49 days; Fig. 5B). It is unclear from our growth data if the observed persistence of WH8102 in P-deplete conditions is a result of the strain’s capability to persist in nutrient-deplete oligotrophic conditions or if they perhaps can support biotic (via unknown enzymatic pathways) or abiotic conversion of P_t_ or Pn to P_i_. A recent study in *E. coli* demonstrated that in the absence of the two known canonical P_i_ transport-related genes that were sequentially deleted, the Phn uptake system could support growth with P_i_ as the sole P source [38].

## Conclusions

All picocyanobacteria strains encode a well-conserved predicted ABC transporter, PhnDCE, that has long been thought to provide a competitive advantage in nutrient deplete conditions. We (for *Synechococcus* isolates) and others (for *Prochlorococcus* isolates) have successfully shown that the PhnD1 protein has a high-affinity for P_t_ and a medium-affinity for P_i_ and MPn. However, most picocyanobacterial strains lack known phosphite degradation or C-P lyase pathways to metabolise P_t_ or MPn. Our findings show that the PhnD1 expression and abundance in the global oceans is not influenced by the phosphate concentration. We further demonstrate the inability of several picocyanobacterial strains (including *Synechococcus* WH8102 and MITS9220 in this study) to grow on P_t_ or MPn as a sole P source. Taken together, these findings suggest that the PhnDCE system may function as a constitutive P_i_ transporter.

None of the *Synechococcus* PhnD1 proteins under investigation displayed a measurable affinity for hypophosphite, implying the requirement for minimally three covalently bound oxygen atoms in a trigonal arrangement to engage specific protein sidechains. Our structure of MITS9220_PhnD1 in complex with P_i_ shows an extensive H-bond network with P_i_. However, they are fewer than the hydrogen bond interactions in the high-affinity PstS protein with P_i_ (e.g. in *E. coli*). This explains the lower measured affinity of picocyanobacterial PhnD1 proteins to P_i_ with a broader substrate range, which may be fortuitous.

We propose two potential scenarios that explain the critical role of PhnD1 in the environment. First, it is likely that PhnD1 aids in the recycling of phospholipid polar headgroups via a predicted phospholipase encoded by *tesA*, which is located adjacent to the *phnDCE* genes. TesA could potentially hydrolyse the commonly found picocyanobacterial phospholipid (PG) to diacylglycerol and glycerol-3-phosphate. Subsequently, the periplasmic alkaline phosphatase would release P_i_, which can then be taken up by the PhnD_1_CE (or Pst) transporter, providing a mechanism for recycling P from the phospholipid bilayer. The second scenario is based on the rate-affinity trade-off that cells mitigate by having multiple transporters for the same nutrient. A medium-affinity P_i_ transporter such as the PhnD_1_CE, with potentially fast transport rates, could help picocyanobacterial cells to acquire large amounts of P_i_ when it is transiently available. This would enable picocyanobacterial cells to balance their requirement for quick transport during sporadic abundance with efficient utilisation of P resources (via Pst transport system) under typical phosphate-deplete conditions.

## Materials and methods

### Genomic and Phylogenetic analyses

Phylogenetic analysis of gene cluster CK_860 annotated as PhnD1 (phosphate/phosphonate-binding proteins) within the Cyanorak database (www.sb-roscoff.fr/cyanorak) [6, 20], was performed using a modified method given by Wilding et al. [39]. Briefly, the 97 orthologous PhnD1 sequences, as well as PhnD2 (CK_6203, 10 sequences) and PhnD3 (CK_56876, 1 sequence), were used to compute a multiple sequence alignment using the L-iNS-I option of MAFFT [40]. The phylogenetic tree was inferred using IQ-Tree [41], using the -TESTONLY option, found to be WAG + G4. The final phylogenetic tree was generated from the inferred and visualised using iTOL [42].

The geographic distribution and expression of PhnD1 and PhnD2 homologues in marine picocyanobacterial populations were analysed using the metagenomes and metatranscriptomes available from the Ocean Microbial Reference Gene Catalogue (OM-RGC) [21, 22]. The protein sequence of *Prochlorococcus* MIT9301_PhnD1 and MIT9301_PhnD2 were used to search against the ‘OM_RGC_v2_metaG’ and ‘OM_RGC_v2_metaT’ catalogue using the default parameters. To limit our analyses to picocyanobacterial sequences, we chose a high significance E-threshold (>e^−75^) and ensured that the homologue matches (with sequence identity >70%) for the PhnD1 and PhnD2 sequences did not overlap. Plots were viewed using P as the environmental variable of interest. Additionally, for comparison, we provide environmental abundance maps for *Prochlorococcus* and *Synechococcus* lineage-specific genes. The protein sequence of a predicted chlorophyll b synthase, PcCAO (CK_2331), specific to *Prochlorococcus* strains and a predicted phycocyanin lyase, CpcS (CK_1523), specific to *Synechococcus* strains were used to search against the ‘OM_RGC_v2_metaG’ catalogue using the default parameters with a high significance E-threshold (>e^−75^).

### Recombinant expression and protein purification

The protein sequences of CC9311_01670, CC9605_01294, MITS9220_01173, and WH8102_01170, annotated to encode PhnD1, were analysed using the SignalP 4.0 [43].

Following truncation of the N-terminal signal peptide (Supplementary Table S3), the genes were PCR-amplified from respective genomic DNA (extracted using the CTAB/phenol-chloroform method) incorporating vector-specific (pOPINF) [44] overhang regions for heterologous expression in *E. coli*. Ligation-independent cloning (Clontech) [45] into the pOPINF vector was carried out using *Kpn*I and *Hin*dIII restriction sites to incorporate an N-terminal hexahistidine tag with a 3C protease cleavage site.

Following plasmid transformation in *E. coli*, all target proteins were expressed to high density using the autoinduction method [46] in a 1 L culture medium. While CC9311_PhnD1, MITS9220_PhnD1, and WH8102_PhnD1 proteins displayed the highest expression in an *E. coli* Lemo strain (BL21 derivative, NE Biolabs), CC9605_PhnD1 achieved higher expression in BL21 derivative strain (Rosetta 2, Novagen). All four PhnD1 proteins were purified using IMAC, as described earlier [47]. Protein-containing fractions were pooled and desalted using size exclusion chromatography (SEC) with a Superdex HiLoad 200 16/600 column (GE Healthcare) equilibrated in buffer containing HEPES (50 mM, pH 7.4), NaCl (300 mM), and glycerol (5% v/v). All buffers contained the reducing agent tris(2-carboxyethyl)phosphine (TCEP, 0.5 mM).

For CC9605_PhnD1, MITS9220_PhnD1, and WH8102_PhnD1 proteins, SEC traces show a single, well-defined peak corresponding to a monomeric state at expected molecular size (~30 kDa). However, for the CC9311_PhnD1 protein, multiple peaks were observed during SEC. Isolation of pure CC9311_PhnD1 occurred by fractionating the peak corresponding to the monomeric species, allowing >90% purity.

### PhnD1 ligand screening and determination of binding affinity to P sources

Ligand screening was performed using differential scanning fluorimetry (DSF) [48] with SYPRO Orange dye (Invitrogen) used to monitor fluorescence at 590 nm following excitation at 485 nm. DSF measures the thermal stability of the protein-ligand complexes, whereby the temperature at which the protein denatures (melting temperature T_*M*_) is shifted to a higher temperature in the presence of a stabilising ligand. The compounds that stabilise the protein the most can then be identified as putative ligands for further binding affinity analysis. Compounds tested incorporated cocktails of common protein stabilising ligands (HR2-096 Silver Bullets, Hampton Research) and single-molecule P sources: phosphate, phosphite, hypophosphite, and methyl phosphonate (each as sodium salts). Each condition was tested in triplicate, with each plate containing a control well with no additive. Thermal melt curves were analysed using the analysis template provided [48] and fitting of Boltzmann distribution (GraphPad Prism) to determine mid-point thermal melt temperatures. Cocktail conditions leading to ≥5 °C in melting temperature (T_*M*_) were considered a significant increase and repeated in triplicate. The thermal melting curve for all four PhnD proteins, CC9311_PhnD, CC9605_PhnD, MITS9220_PhnD and WH8102_PhnD, showed a single transition point with a melting temperature (T_*M*_) of 47 °C, 32 °C, 46 °C, and 35 °C, respectively.

To elucidate the affinity of PhnD1 to P, the DSF assay was modified and carried out with increasing ligand concentrations as previously described [47], leading to an incremental shift in observed melting temperatures. Data were processed using the provided Python package [29]. Results were plotted and checked for consistency by independently determining EC_50_ values (GraphPad Prism). The standard error of derived K_*D*_ values was determined by comparing the deviation in fitting three individual replicates. It was, in all cases, smaller than 10% of the determined affinity measurement.

### MITS9220_PhnD1 crystallisation and structure determination

All four *Synechococcus* PhnD1 purified protein products were subjected to sparse matrix crystallisation screening at 20 °C by sitting drop vapour diffusion method by mixing 0.1 μL of protein (10 mg/mL) with 0.1 μL of the reservoir. However, only MITS9220_PhnD1 showed good diffraction-quality crystals grown in 0.1 M Tris pH 8.5, 25% PEG 3350. Before plunge freezing in liquid nitrogen, MITS9220_PhnD1 crystals were cryo-protected in the mother liquor with an additional 30% (v/v) ethylene glycol. The crystals were further transferred into the vacuum vessel using an adapted cryotransfer system (Leica VCT100).

The data were collected at the Diamond Light Source I23 beamline [49], equipped with the semi-cylindrical Pilatus 12 M (Dectris AG, Switzerland) detector, at four different wavelengths, 2.7552, 3.0996, 4.8621 and 5.1666 Å. Data were processed using XDS [50] and merged using XSCALE. The structure of MITS9220_PhnD1 was solved by experimental phasing using the SAD dataset collected at 3.0996 Å wavelength using the CRANK2 pipeline [51]. After density modification, automated model building within the CRANK2 pipeline produced an initial model comprising a single copy. Several rounds of manual model building and refinement were carried out in COOT [52]and Refmac5 [53] before validating the final model with Molprobity [54]. The final coordinates contain residues 1–270 of the SignalP truncated mature sequence in one chain, with one defined phosphate site, nine chloride anions and three EDO molecules. Final refinement statistics are given in Table 2, and coordinates are deposited in the PDB (7S6G). Anomalous difference Fourier from long-wavelength data was generated using ANODE [55]. The positions of anomalous peaks higher than 4.0σ as output by ANODE from datasets both above (2.755 Å) and below the Ca-edge (3.0996 Å) were inspected in COOT [52] to discount these peaks as calcium. The peaks at 3.0996 Å combined with their absence at 4.8621 Å are indicative of chloride ions. To ascertain whether a phosphate or sulphate was present in the ligand site, anomalous difference Fourier maps were compared from data collected above (4.8621 Å) and below (5.1666 Å) the sulphur absorption edge. The strong peak present in both datasets suggests that the ligand contains phosphorus (Supplementary Fig. S8).

### *Synechococcus* MITS9220 and WH8102 growth in the presence of alternate P sources

*Synechococcus* MITS9220 and WH8102 cultures were grown at 22 °C, with 40 µM photons m^−2^ s^−1^ continuous illumination, in an orbital shaker at 100 rpm in acid-washed polycarbonate flasks using Red Sea salt-based PCR-S11 medium [56]. For treatment conditions containing alternative P compounds, 50 µM NaH_2_PO_4_ was replaced with 50 µM phosphite (Na_2_HPO_3_·5H_2_O) or methylphosphonate. In P-deplete media, an equal volume of water was added instead of a P compound. Experimental cultures were started from mid-logarithmic cultures grown in phosphate-containing PCR-S11 media. A small inoculum (~100 µL) was used to minimise the transfer of phosphate from stock cultures. Four biological replicates were tested for each experimental condition. Growth was regularly monitored via cell counts using a CytoFLEX S flow cytometer. Cells were identified by chlorophyll and phycoerythrin fluorescence the following excitation using a blue laser (488 nm). Phosphate concentration in all treatment conditions was monitored regularly for the duration of the experiments using a Phosphate Colorimetric kit following the manufacturer’s instructions (Sigma-Aldrich) with a detection limit of 0.5 µM.

## Supplementary information

Supplementary figures

Supplementary tables

## Data Availability

The structure coordinates for MITS9220_PhnD1 are deposited in the Protein Data Bank (accession code 7S6G). All other study data is included in the article and/or supporting information files.
