# Supplementary material for: Marine picocyanobacterial PhnD1 shows specificity for various phosphorus sources but likely represents a constitutive inorganic phosphate transporter
Source: ISME J. 2023 Apr 22;17(7):1040–51. doi: 10.1038/s41396-023-01417-w (PMC10284923; doi:10.1038/s41396-023-01417-w)
Supplement: Supplementary file 1 — Supplementary figures [file 41396_2023_1417_MOESM1_ESM.pdf]

Supplementary information

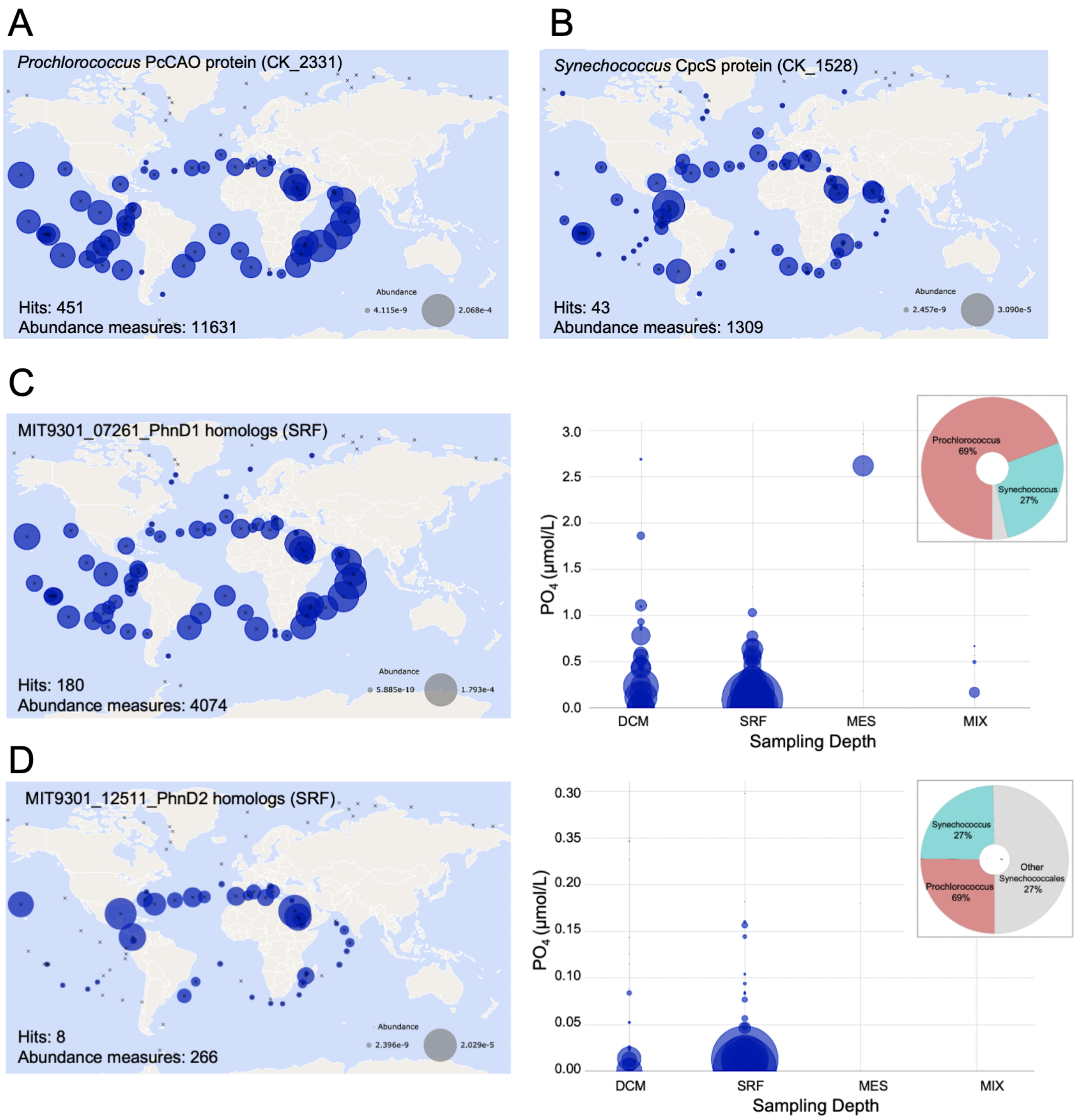

Figure S1: Ocean metagenome abundance of picocyanobacterial PhnD1 and PhnD2 sequences

The environmental abundance for **(A)** *Prochlorococcus* PcCAO protein **(B)** *Synechococcus* CpcS protein **(C)** *Prochlorococcus* MIT9301_PhnD1 and **(D)** MIT9301_PhnD2 picocyanobacterial homologs extracted from the Tara Oceans MetaG dataset [50]. Abundance is plotted for surface waters, with a circle size corresponding to the measured abundance at a particular sampling site. Sampling sites are denoted by an 'X'. The corresponding bubble plot (right) for the identified sequences across sampling depths (SRF, surface waters; DCM, deep chlorophyll maximum; MES, mesopelagic zone; MIX, marine epipelagic mixed layers;) is depicted as a function of measured phosphate concentration. The Krona plot in the inset shows the taxonomic distribution of MIT9301_PhnD1, and MIT9301_PhnD2 homolog hits selected to analyse the metagenome abundance.

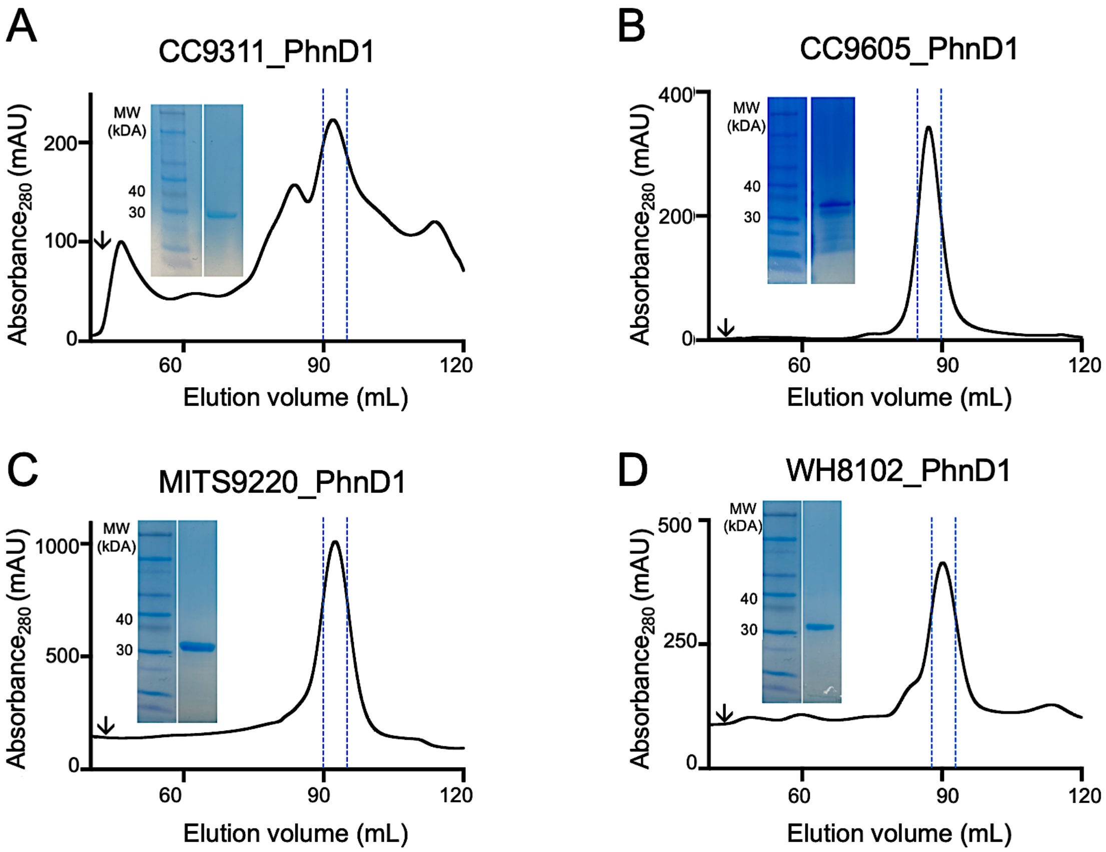

Figure S2: Preparative size exclusion traces of four *Synechococcus* PhnD1 proteins

From top left: CC9311_PhnD, CC9605_PhnD, MITS9220_PhnD, and WH8102_PhnD with dashed lines indicating collected protein eluate. Purified fraction, as analysed using SDS-PAGE, is shown inset for each purified protein. Void volume (V_0) is marked with an arrow for all traces. Preparative SEC was performed using a Superdex 200 HiLoad 16/600 column (GE Healthcare) equilibrated in HEPES buffer (50 mM) with NaCl (300 mM) and glycerol (5% v/v) on an ÄktaPure FPLC system operating at a flow rate of 1 mL min^{-1}.

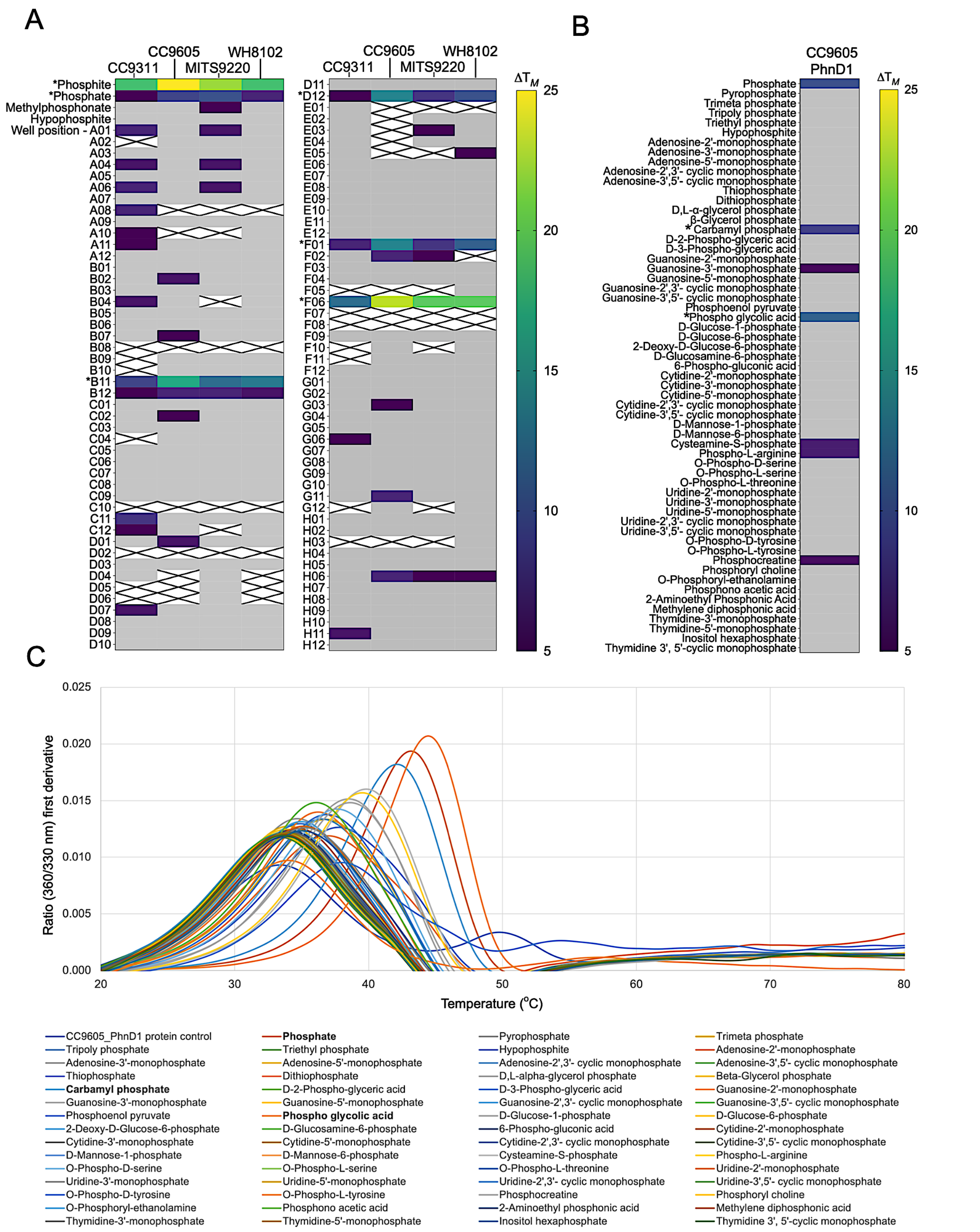

Figure S3: Ligand screening of *Synechococcus* PhnD1 proteins

(A) Heat map of the DSF thermal melt assays of *Synechococcus* PhnD1 proteins in the presence of a range of cocktail solutions (Silver Bullets 96-well screen), showing a significant change in the melting temperature (ΔT_M). **(B)** For CC9605_PhnD1, 60 additional P-sources were screened via DSF, the ΔT_M for these conditions are depicted in the heatmap. **(C)** The thermal melt curves of CC9605_PhnD1 in the presence of each condition are additionally provided.

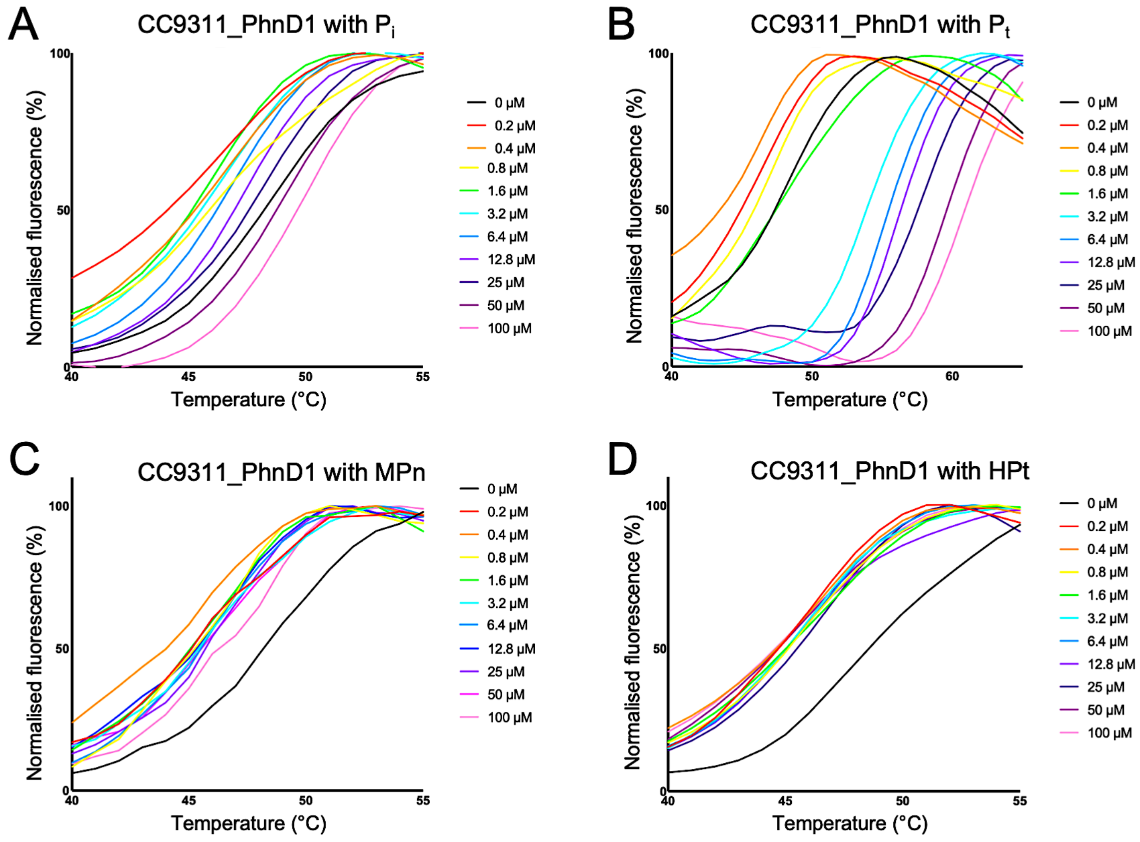

Figure S4: Fluorometric isothermal binding data for CC9311_PhnD1

Thermal shifts for CC9311_PhnD1 protein are shown in increasing concentrations of **(A)** sodium phosphate, **(B)** sodium phosphite, **(C)** methyl phosphonate, and **(D)** sodium hypophosphite. All reactions were performed in 20 μL format containing 5 μM of purified protein and identical buffer (HEPES (50 mM, pH 7.4), NaCl (300 mM), glycerol (5% v/v).

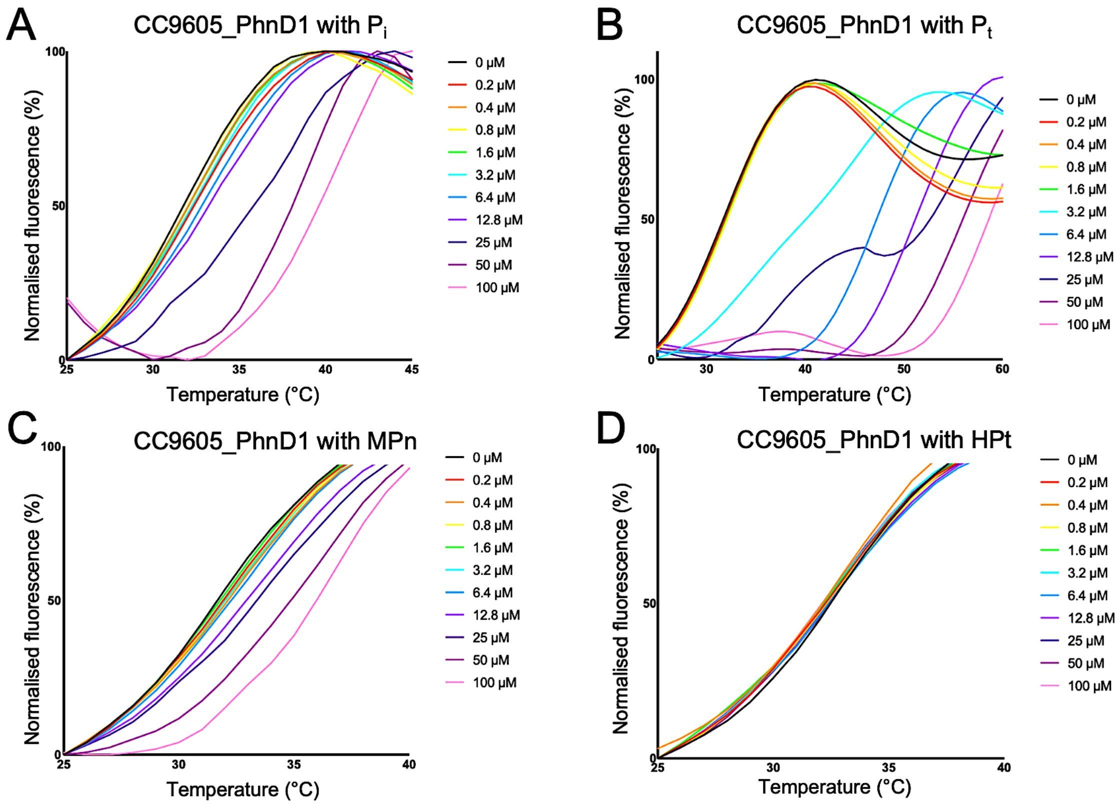

Figure S5: Fluorometric isothermal binding data for CC9605_PhnD1

Thermal shifts for CC9605_PhnD1 protein are shown in increasing concentrations of **(A)** sodium phosphate, **(B)** sodium phosphite, **(C)** methyl phosphonate, and **(D)** sodium hypophosphite. All reactions were performed in 20 μL format containing 5 μM of purified protein and identical buffer (HEPES (50 mM, pH 7.4), NaCl (300 mM), glycerol (5% v/v).

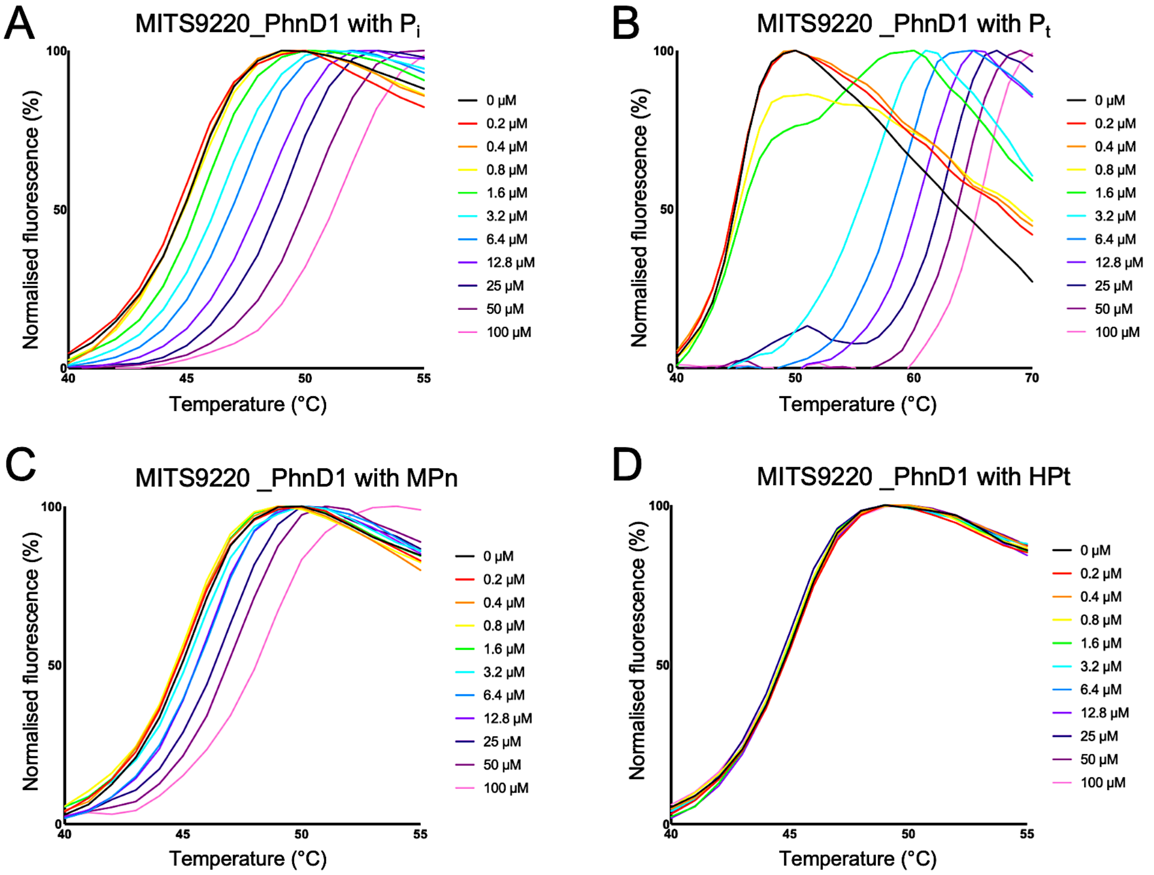

Figure S6: Fluorometric isothermal binding data for MITS9220_PhnD1

Thermal shifts for MITS9220_PhnD1 protein are shown in increasing concentrations of **(A)** sodium phosphate, **(B)** sodium phosphite, **(C)** methyl phosphonate, and **(D)** sodium hypophosphite. All reactions were performed in 20 μL format containing 5 μM of purified protein and identical buffer (HEPES (50 mM, pH 7.4), NaCl (300 mM), glycerol (5% v/v).

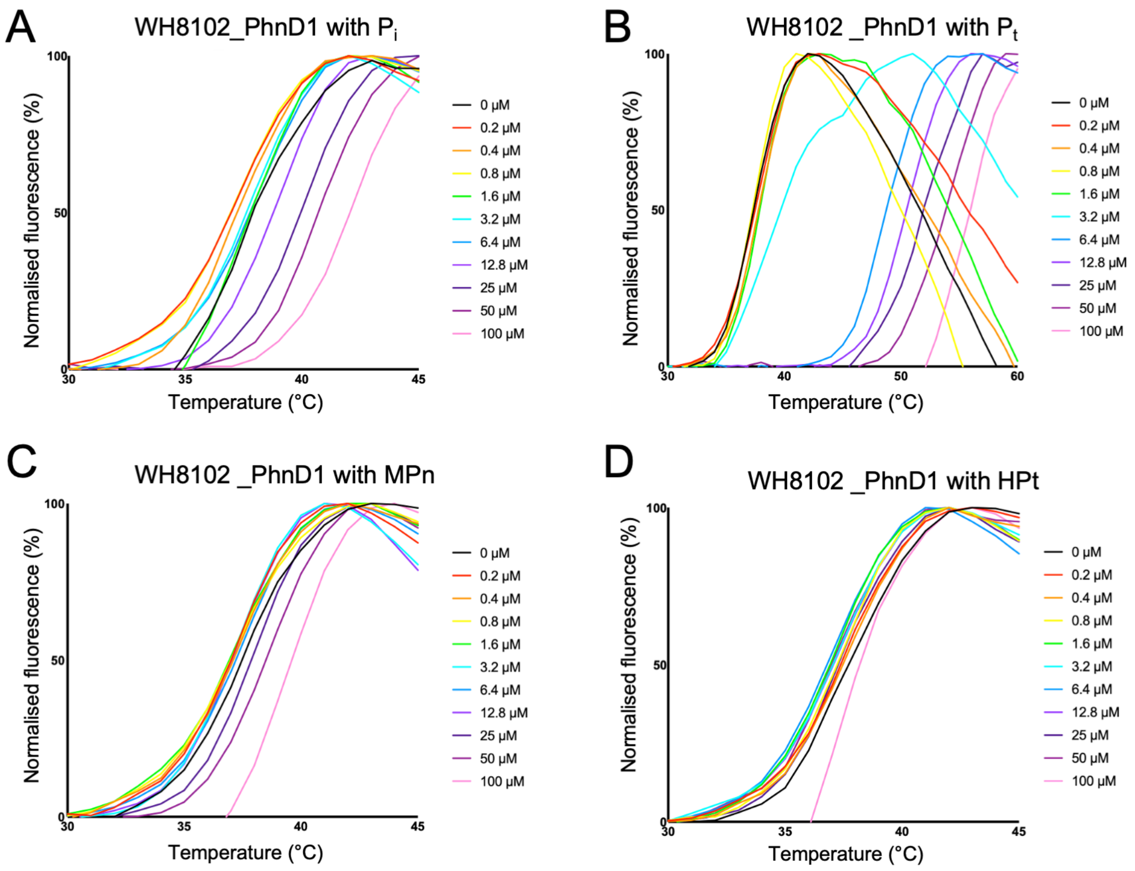

Figure S7: Fluorometric isothermal binding data for WH8102_PhnD1

Thermal shifts for WH8102_PhnD1 protein are shown in increasing concentrations of **(A)** sodium phosphate, **(B)** sodium phosphite, **(C)** methyl phosphonate, and **(D)** sodium hypophosphite. All reactions were performed in 20 μL format containing 5 μM of purified protein and identical buffer (HEPES (50 mM, pH 7.4), NaCl (300 mM), glycerol (5% v/v).

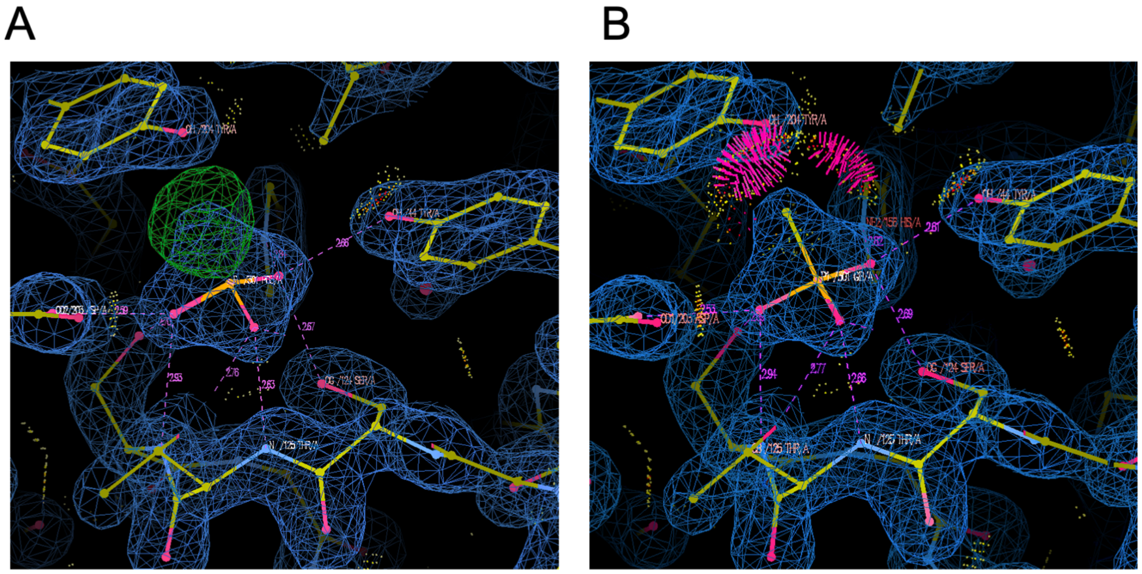

Figure S8: Synechococcus MITS9220_PhnD1 ligand validation

The post-refinement $2F_0$-F_c electron density maps (blue mesh, contoured at 2σ) and F_0-F_c difference maps (green mesh, contoured at 4σ) generated during the validation of ligand binding. In **(A),** a phosphite moiety is modelled and refined into the MITS9220_PhnD1 ligand density, producing a large positive peak (green mesh) on the R1 oxygen position. In **(B)**, a methyl phosphonate (MPn) moiety is modelled and refined into the MITS9220_PhnD1 ligand density, showing a large steric clash (pink spike patches) with the adjoining Y204 capping residue.

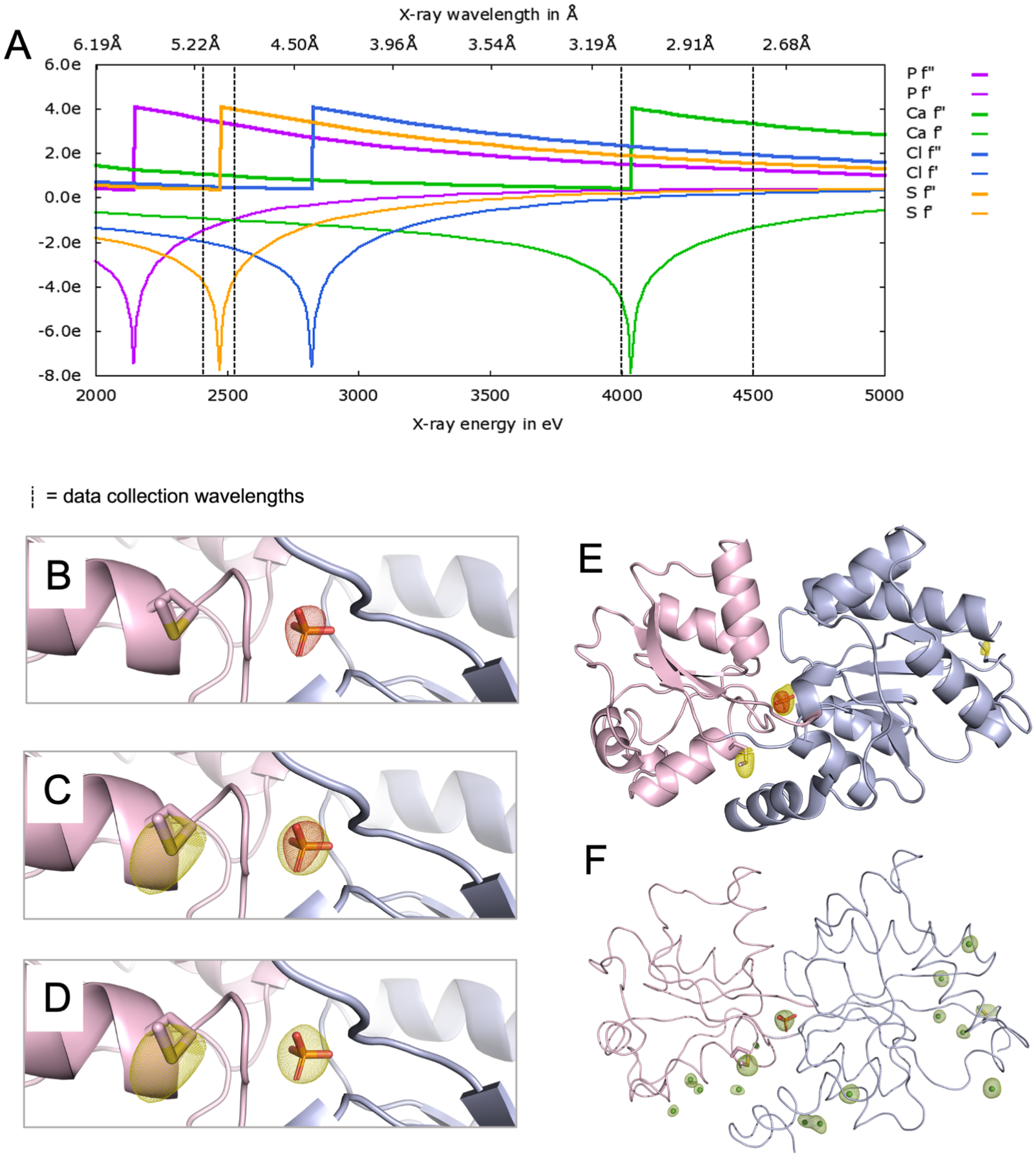

Figure S9: Anomalous absorption data collected for MITS9220_PhnD1 crystal
(A) Anomalous absorption plot of P, Ca, Cl and S over the energy range 2000eV to 5000eV, between which data were collected at the I23 beamline for MITS9220_PhnD1 crystal. Dashed lines show the energy at which data was collected to identify elements present within the anomalous difference maps. **(B)** Close-up of P_i and methionine residue at 2400eV with anomalous difference Fourier map in red (4σ) **(C)** Close-up of P_i and methionine residue at 2400eV with anomalous difference Fourier map in red (4σ) and 2550eV anomalous difference Fourier map in yellow (4σ). **(D)** Close-up of P_i and methionine residue at 2550eV with anomalous difference Fourier map in yellow (4σ). **(E)** Cartoon overview of the MITS9220_PhnD1 structure with 2400eV anomalous difference Fourier map in red (4σ) and 2550eV anomalous difference Fourier map in yellow (4σ). **(F)** Ribbon overview of the MITS9220_PhnD1 structure with 4000eV anomalous difference Fourier map in green (4σ) highlighting positions of P_i, sulphurs from methionine residues and Cl ions (green sphere).
