# Supplementary material for: Marine picocyanobacterial PhnD1 shows specificity for various phosphorus sources but likely represents a constitutive inorganic phosphate transporter
Source: ISME J. 2023 Apr 22;17(7):1040–51. doi: 10.1038/s41396-023-01417-w (PMC10284923; doi:10.1038/s41396-023-01417-w)
Supplement: Supplementary file 2 — Supplementary tables [file 41396_2023_1417_MOESM2_ESM.pdf]

Table S1: Silver bullet screen used for PhnD1 ligand screening

	1	2	3	4	5	6	7	8	9	10	11	12
A	0.33% w/v 1,5-Naphthalenedisulfonic acid disodium salt/2,5-Pyridinedicarboxylic acid/3,5-Dinitrosalicylic acid	0.25% w/v Benzidine/Nicotinamide/Pyromellitic acid/Sulfaguanidine	0.25% w/v Gly-gly/Gly-gly-gly/Gly-gly-gly-gly/Pentaglycine	0.25% w/v 3,5-Dinitrosalicylic acid/4-Aminobenzoic acid/Salicylic acid/Trimesic acid	0.33% w/v 4-Nitrobenzoic acid/5-Sulfosalicylic acid dihydrate/Naphthalene-1,3,6-trisulfonic acid trisodium salt hydrate	0.33% w/v 2,6-Naphthalenedisulfonic acid disodium salt/2,7-Naphthalenedisulfonic acid disodium salt/Anthraquinone-2,6-disulfonic acid disodium salt	0.33% w/v 1,5-Naphthalenedisulfonic acid disodium salt/Naphthalene-1,3,6-trisulfonic acid trisodium salt hydrate/PIPES	0.25% w/v 3,5-Dinitrosalicylic acid/3-Aminosalicylic acid/Salicylamide/Sodium 1-pentanesulfonate monohydrate	0.16% w/v L-Histidine/L-Isoleucine/L-Leucine/L-Phenylalanine/L-Tryptophan/L-Tyrosine	0.20% w/v D-(+)-Trehalose dihydrate/Guanidine hydrochloride/Phenol/Trimethylamine N-oxide dihydrate/Urea	0.33% w/v 2,5-Pyridinedicarboxylic acid/4-Nitrobenzoic acid/Mellitic acid	0.25% w/v Benzidine/Phenylglyoxal monohydrate/Sulfaguanidine/Sulfanilamide
B	Control	0.33% w/v 1,3,5-Pentanetricarboxylic acid/5-Sulfosalicylic acid dihydrate/Trimesic acid	0.25% w/v 5-Sulfoisophthalic acid monosodium salt/Cystathionine/Dithioerythritol/L-Citrulline	0.33% w/v 3,5-Dinitrosalicylic acid/3-Aminobenzenesulfonic acid/5-Sulfosalicylic acid dihydrate	0.33% w/v 2,7-Naphthalenedisulfonic acid disodium salt/Azelaic acid/trans-Cinnamic acid	0.33% w/v 2,6-Naphthalenedisulfonic acid disodium salt/2-Aminobenzenesulfonic acid/m-Benzenedisulfonic acid disodium salt	0.33% w/v 1,4-Cyclohexanedicarboxylic acid/2,2'-Thiodiglycolic acid/5-Sulfoisophthalic acid monosodium salt	0.33% w/v 3-Aminobenzoic acid/3-Aminosalicylic acid/Salicylic acid	0.25% w/v Hexamminecobalt(III) chloride/Salicylamide/Sulfanilamide/Vanillic acid	0.25% w/v p-Coumaric acid/Phenylurea/Poly(3-hydroxybutyric acid)/Sulfaguanidine	0.25% w/v 1,2-Diaminocyclohexane sulfate/1,4-Cyclohexanedicarboxylic acid/Methylenediphosphonic acid/Sulfanilic acid	0.25% w/v D-Fructose 1,6-bisphosphate trisodium salt hydrate/D-Glucose 6-phosphate sodium salt/L-O-Phosphoserine/O-Phospho-L-tyrosine
C	0.25% w/v Benzamidine hydrochloride/L-Carnitine hydrochloride /L-Cystine/L-Ornithine hydrochloride	0.33% w/v Caffeine/Dithioerythritol/L-Methionine	0.25% w/v Ala-Ala/Ala-gly /Gly-gly-gly-gly/Leu-gly-gly	0.20% w/v Aspartame/Gly-asp/Gly-ser/Ser-tyr /Tyr-phe	0.16% w/v Ala-ala/Aspartame/Gly-tyr/Leu-gly-gly/Ser-glu/Tyr-ala	0.33% w/v Gly-phe/Gly-tyr/Leu-gly-gly	0.16% w/v Ala-ala/Gly-asp/Gly-gly/Gly-phe/Gly-ser/Ser-tyr	0.05% w/v Glycine/L-(-)-Threonine/L-(+)-Lysine/L-Alanine /L-Arginine/L-Asparagine monohydrate/L-Aspartic acid/L-Glutamic acid/L-Glutamine/L-Histidine/L-Isoleucine/L-Leucine/L-Methionine/L-Phenylalanine/L-Proline/L-Serine/L-Tryptophan/L-Tyrosine/L-Valine	0.20% w/v D-(+)-Maltose monohydrate /D-(+)-Melibiose monohydrate/D-(+)-Raffinose pentahydrate/D-(+)-Trehalose dihydrate/Stachyose hydrate	0.16% w/v β-Cyclodextrin/D-(+)-Cellobiose/D-(+)-Maltotriose /D-(+)-Melezitose hydrate /D-(+)-Raffinose pentahydrate/Stachyose hydrate	0.16% w/v Azelaic acid/m-Benzenedisulfonic acid disodium salt/Mellitic acid/Pimelic acid/Pyromellitic acid/trans-Cinnamic acid	0.25% w/v 5-Sulfoisophthalic acid monosodium salt/Anthraquinone-2,6-disulfonic acid disodium salt/N-(2-acetamido)-2-aminoethanesulfonic acid/Tetrahydroxy-1,4-benzoquinone hydrate
D	0.25% w/v 1,3,5-Pentanetricarboxylic acid/5-Sulfosalicylic acid dihydrate/o-Sulfobenzoic acid monoammonium salt/Sodium 4-aminosalicylate dihydrate	0.06 M CHAPS/HEPES/Tris/ 0.25% (w/v) Hexamminecobalt(III) chloride	0.06 M MES monohydrate/PIPES/ 0.33% (w/v) Hexamminecobalt(III) chloride	0.005 M Gadolinium(III) chloride hexahydrate/Samarium(III) chloride hexahydrate/ 0.05M Benzamidine hydrochloride/ 0.25% (w/v) Salicin	0.004 M Calcium chloride dihydrate/Magnesium chloride hexahydrate/Manganese(II) chloride tetrahydrate/Zinc chloride	0.004 M Cadmium chloride hydrate/Cobalt(II) chloride hexahydrate/Copper(II) chloride dihydrate/Nickel(II) chloride hexahydrate	0.25% w/v 3,5-Dinitrosalicylic acid/3-Indolebutyric acid/Naphthalene-1,3,6-trisulfonic acid trisodium salt hydrate/trans-1,2-Cyclohexanedicarboxylic acid	0.20% w/v Betaine anhydrous/L-Glutamic acid/L-Proline/Taurine/Trimethylamine N-oxide dihydrate	0.25% w/v 1,2-Diaminocyclohexane sulfate/4-Nitrobenzoic acid/Cystamine dihydrochloride/Spermine	0.25% w/v 1,5-Naphthalenedisulfonic acid disodium salt/2,7-Naphthalenedisulfonic acid disodium salt/5-Sulfoisophthalic acid monosodium salt/Sulfanilic acid	0.25% w/v 2,6-Naphthalenedisulfonic acid disodium salt/4-Aminobenzoic acid/5-Sulfosalicylic acid dihydrate/Naphthalene-1,3,6-trisulfonic acid trisodium salt hydrate	0.20% w/v Rhenium(IV) oxide/Sodium bromide/Sodium nitrate /Sodium phosphate dibasic dihydrate /Sodium tetraborate decahydrate
E	0.20% w/v Caffeine/Cytosine/Gallic acid monohydrate/Nicotinamide/Sodium pyrophosphate tetrabasic decahydrate	1% w/v Digest of Dextran sulfate with α-Amylase and Dextranase/Dextran sulfate sodium salt/ (0.005% w/v) Dextranase/α-Amylase	1% w/v Tryptone	1% w/v Protamine sulfate	/ Digest of Ribonucleic acid and Deoxyribonucleic acid with Ribonuclease A and Deoxyribonuclease I/ (0.005% w/v) Deoxyribonuclease I & Ribonuclease A/(0.5% w/v) Deoxyribonucleic acid & Ribonucleic acid	0.5% w/v Digest of Casein and Hemoglobin with Pepsin, Trypsin, Protease and Proteinase K/Casein/Hemoglobin/ (0.005% w/v) Pepsin/Protease/Proteinase K/Trypsin	Control	0.20% w/v D-Sorbitol/GlycerolGlycinemyo-Inositol/Sarcosine	0.20% w/v 1,4-Diaminobutane/Cystamine dihydrochlorideDiloxanide furoateSarcosine/Spermine	0.25% w/v 1,2-Diaminocyclohexane sulfate/1,8-Diaminooctane/Cadaverine/Spermine	0.20% w/v 1,2-Diaminocyclohexane sulfate/Diloxanide furoateFumaric acidSpermine/Sulfaguanidine	0.20% w/v 1,4-Diaminobutane/1,8-Diaminooctane/Cadaverine/Cystamine dihydrochloride/Spermidine
F	0.25% w/v Methylenediphosphonic acid/Phytic acid sodium salt hydrate/Sodium pyrophosphate tetrabasic decahydrate/Sodium triphosphate pentabasic	0.20% w/v D-Fructose 1,6-bisphosphate trisodium salt hydrate/Glycerol phosphate disodium salt hydrate/L-O-PhosphoserineO-Phospho-L-tyrosine/Phytic acid sodium salt hydrate	0.16% w/v 4-Aminobutyric acid/6-Aminohexanoic acidL-(+)-LysineL-Ornithine hydrochloride/Taurine/β-Alanine	0.20% w/v L-Arginine/L-CanavanineL-Carnitine hydrochloride L-Citrulline/Taurine	0.20% w/v 1,2,3-Heptanetriol/1,3-Propanediol1,6-HexanediolGly-gly/Resorcinol	0.20% w/v (±)-2-Methyl-2,4-pentanediol/1,2,3-Heptanetriol Diethylenetriamine pentakis(methylphosphonic acid) D-Sorbitol/Glycerol	0.20% w/v Barbituric acid/Betaine anhydrous/Phloroglucinol/Resorcinol/Tetrahydroxy-1,4-benzoquinone hydrate	0.20% w/v 1,6-Hexanediol/Diethylenetriaminepentakis(methylphosphonic acid)/Gly-glymyo-Inositol/Phloroglucinol	0.20% w/v 6-Aminohexanoic acid/Benzamidine hydrochloride/Congo Red/Nicotinamide/Salicin	0.20% w/v Anthrone/Benzidine/N-(2-acetamido)-2-aminoethanesulfonic acid/Phenylurea/β-Alanine	0.25% w/v 4-Aminobutyric acid/CytosineSalicylamide/Sodium 1-pentanesulfonate monohydrate	0.11% w/v Dodecanedioic acid/Fumaric acid/Glutaric acid/Hexadecanedioic acid/Maleic acid/Oxamic acid/Pimelic acid/Sebacic acid/Suberic acid
G	0.16% w/v 5-Sulfosalicylic acid dihydrate/Dodecanedioic acid/Hippuric acid/Mellitic acid/Oxalacetic acid/Suberic acid	0.20% w/v 2,2'-Thiodiglycolic acid/Adipic acid/Benzoic acid/Oxalic acid anhydrous/Terephthalic acid	0.25% w/v 2,2'-Thiodiglycolic acid/Azelaic acid/Mellitic acid/trans-Aconitic acid	0.16% w/v 3-Indolebutyric Acid/Hexadecanedioic acid/Oxamic acid/Pyromellitic acid/Sebacic acid/Suberic acid	0.25% w/v 1,3,5-Pentanetricarboxylic acid/4-Hydroxyphenylacetic acid/Benzoic acid/Poly(3-hydroxybutyric acid)	0.16% w/v Glutaric acid/Mellitic acid/Oxalic acid anhydrous/Pimelic acid/Sebacic acid/trans-Cinnamic acid	0.20% w/v 4-Aminobenzoic acid/Azelaic acido-Sulfobenzoic acid monoammonium salt/p-Coumaric acid/Sodium 4-aminosalicylate dihydrate	0.16% w/v 3-Aminobenzenesulfonic acid/3-Aminobenzoic acidHippuric acid/Oxalacetic acid/Salicylic acid/Trimesic acid	0.20% w/v 2-Aminobenzenesulfonic acid/3-Indolebutyric acid/4-Hydroxyphenylacetic acid/Barbituric acid/Terephthalic acid	0.20% w/v 1,4-Cyclohexanedicarboxylic acid/2,5-Pyridinedicarboxylic acidGlutaric acidtrans-1,2-Cyclohexanedicarboxylic acid/trans-Aconitic acid	10% v/v TACSIMATE pH 7.0	0.20% w/v Benzenephosphonic acid/Gallic acid monohydrate/MelatoninN-(2-carboxyethyl)-iminodiacetic acid/Trimellitic acid
H	0.20% w/v 1,3-Propanediol/D-3-Phosphoglyceric acid disodium salt/Gly-glyGlycerol phosphate disodium salt hydrate/Maleic acid	0.20% w/v Ala-ala/Ala-gly Gly-asp/Gly-phe/Ser-glu	0.20% w/v 3,5-Dinitrosalicylic acid/4-Aminobenzoic acid/Benzamidine hydrochloride/Hexamminecobalt(III) chloride/Mellitic acid	0.16% w/v 1,4-Diaminobutane/1,8-Diaminooctane/Cadaverine/Cystamine dihydrochloride/Spermidine/Spermine	0.16% w/v 4-Aminobutyric acid/6-Aminohexanoic acid/Oxamic acidSulfanilic acid/Trimesic acid/β-Alanine	0.16% w/v D-3-Phosphoglyceric acid disodium salt/D-Fructose 1,6-bisphosphate trisodium salt hydrate/D-Glucose 6-phosphate sodium salt/L-O-Phosphoserine/O-Phospho-L-tyrosine/Phytic acid sodium salt hydrate	0.0625% w/v 1,3,5-Pentanetricarboxylic acid/Azelaic acid/Dodecanedioic acid/Glutaric acid/Hexadecanedioic acid/Pimelic acid/Sebacic acid/Suberic acid	0.16% w/v 1,5-Naphthalenedisulfonic acid disodium salt/2,6-Naphthalenedisulfonic acid disodium salt/2,7-Naphthalenedisulfonic acid disodium salt/4-Nitrobenzoic acid/m-Benzenedisulfonic acid disodium salt/Naphthalene-1,3,6-trisulfonic acid trisodium salt hydrate	0.2% w/v 2,5-Pyridinedicarboxylic acid/Pyromellitic acid/Salicylic acid/trans-1,2-Cyclohexanedicarboxylic acid/trans-Cinnamic acid	0.16% w/v 3-Aminobenzenesulfonic acid/5-Sulfosalicylic acid/dihydratep-Coumaric acid/PIPES/Terephthalic acid/Vanillic acid	0.07% w/v Barbituric acid/Benzidine/CystathionineL-Canavanine/L-Carnitine hydrochloride/L-Cystine/Mellitic acid	0.16% w/v Aspartame/Gly-gly-glyLeu-gly-gly/Pentaglycine/Tyr-ala/Tyr-phe

Table S2: Hydrogen bonds between phosphate and MITS9220_PhnD1 or *E. coli* PstS

Phosphate atom	MITS9220_PhnD1 (domain location) PDB: 7S6G	Distance (Å)	E.coli_PstS (domain location) PDB: 2ABH	Distance (Å)
O1	S126 OG (II)	2.7	T10 N (I)	2.9
	S126 N (II)	2.9	T10 OG1 (I)	2.7
	D203 OD2 (I)	2.5	R135 NH2 (II)	2.8
O2	Y204 OH (I)	3.1	R135 NH2 (II)	3.3
			R135 NH1 (II)	2.9
			S139 OG (II)	2.8
			T141 OG1 (II)	2.7
			T141 N (II)	2.8
O3	Y44 OH (I)	2.6	S38 N (I)	2.8
	S124 OG (II)	2.7	S38 OG (I)	2.7
	H156 NE2 (II)	2.8	G140 N (II)	2.8
O4	T125 N (II)	2.6	T10 N (I)	3.1
	T125 OG1 (II)	2.8	F11 N (I)	2.8
	Water	2.7	D56 OD2 (I)	2.5

Table S3: Signal P truncated, mature PhnD1 sequences

Target	Sequence
CC9311_PhnD1	LAFLCSQGSAVLPANAQATLRIGAIPDQNPERLNRRYGQLATEL SDKLKVPVRYVPVSNYPAAVSAFRTGSLDLVWFGGLTGVQARLQ TPGAKVLAQRAIDAKFQSVFIANTSAGLKPFSNINGLKGLKGKR FTFGSESSTSGRLMPQHFLAKAGVTPKQFAGGQAGFSGSHDATI ALVQSGSYQAGALNELVWDVAVKKGNVDPTKVKVIWKTPPYVDY HWVARPNLDQRFGKGFTTKLQKAILGLTPSTQRQKTILELFGAK RFIPAQESEYQPIEQVGRQLGKIR
CC9605_PhnD1	APQNDTKQAVLQIGAIPDQNPEKLNRLYGTLSSELSEKLDVPVR YAPVSNYAAAVSAFRTGSLDLVWFGGLTGVQARLQTPGARVLAQ REIDAEFTSVFIANGASGLRPITSGDQLVELKGRRLAFGSESST SGRLMPQFFMGENGVKPEDLAGGGPGFSGSHDATIAVVQSGAYE VGALNEQVWRSNMADGRVDPSKVSVIWRTPPYVDYHWVVRPGLD ERFGDGFTDKLQTALLDLSADTENGATILELFGAERFIPAKDED YVMIETVGRQLGKIR
MITS9220_PhnD1	QPRLKVGAIPDQNPERLNRLYGQLADELSDRLNVKVRYVPVSNY PAAVSAFRTGGLDLVWFGGLTGVQARLQTPGAQVLAQRDIDARF RSVFIANTSSGLQPISSINGLTSLRGKRFSFGSESSTSGRLMPQ HFLAKAGVTPSQFSGGRAGFSGSHDATIAVVQSGAYEAGALNEQ VWTSAVNDGRVNTEKVSVIWRTPEYVDYHWVVRPKLDQRFGKGF TTRLQKAILGLEPTTPRQVTILELFAAKRFIPVEASQYKPIEKV GRELGKIR
WH8102_PhnD1	APPSADQVLRIGAIPDQNPEKLNRLYGSLSDELSDSLNVAVRYV PVSNYAAAVSAFRSGSLDLVWFGGLTGVQARLQTPGAMVLAQRD IDAKFTSVFIANGASGLRPITSADQLVQLKGRRLAFGSESSTSG RLMPQYFLGESGVTMADLAGGAPGFSGSHDATIAVVESGAYEVG ALNEQVWRSNVDEGRVDTDKVAVIWRTAPYVDYHWVARPDLDAR FGKGFTDRVQSSLLSLTPATERGALVLELFGAKRFIPAQNEAYA KIEAVGRQLGKIR
